# Total Synthesis of Penicyclone A Using a Double Grignard Reaction

**DOI:** 10.1021/acs.joc.2c02200

**Published:** 2022-11-16

**Authors:** Gregor Talajić, Edi Topić, Jerko Meštrović, Nikola Cindro

**Affiliations:** Department of Chemistry, Faculty of Science, University of Zagreb, Horvatovac 102a, 10000 Zagreb, Croatia

## Abstract

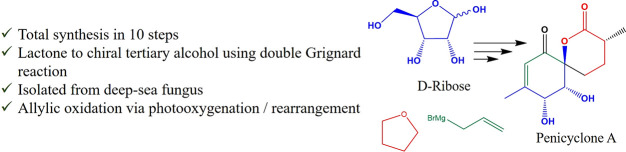

We describe the first total synthesis of penicyclone A, a novel deep-sea fungus-derived polyketide, and a reevaluation of its antimicrobial activity. The synthesis of this unique spirolactone was achieved in 10 steps starting from a known d-ribose derivative. The key steps include a double Grignard reaction for the diastereoselective construction of the chiral tertiary alcohol intermediate, tandem oxidation/cyclization, and photooxygenation, followed by an oxidative rearrangement to introduce the enone functionality.

## Introduction

Penicyclone A is a deep-sea derived natural product containing a spiro[5.5]lactone.^1^ This structural motif is rare with only a few natural products reported to date.^2^ As such, it presents a considerable synthetic challenge owing to the limited scope of methods applicable for the construction of such a moiety. Our motivation for the synthesis of penicyclone A, aside from its exotic structure, was its reported antibacterial activity. Bacterial resistance to current antibiotics in medicinal use presents a significant challenge in health care.^3^ One approach to resolving this challenge is the synthetic modification of existing scaffolds to bypass antimicrobial resistance (AMR). However, derivatives of a compound to which bacteria is already resistant pose a high risk of bacterial adaptation. To alleviate this risk, it is beneficial to explore the antimicrobial activity of completely new scaffolds.^4^ In 2015, Li and coworkers reported the isolation of penicyclones A–E, a family of polyketide secondary metabolites that were harvested from the fungus *Penicillium* sp. F23-2 when the fungal strain was cultured on a rice-based solid medium.^1^ This fungal strain was known for producing cytotoxic nonribosomal peptide synthetases (NRPS) alkaloids and terpenoids in a potato-based medium under static conditions,^5^ as well as nitrogen-containing polyketides (sorbicillinoids) in agitated peptone yeast glucose broth (PYG) medium.^6^ The one-strain-many-compounds (OSMAC) approach along with altering the cultivation conditions resulted in the isolation of these new secondary metabolites. After isolation, the compounds were not only thoroughly characterized, but their minimum inhibitory concentration (MIC) values were also measured to have impressive results for *Staphylococcus aureus*, especially in the case of penicyclone A (0.3 μg/mL). In contrast with NRPS alkaloids, these penicyclone compounds showed no cytotoxic activity toward HeLa, BEL-7402, KEK-293, HCT-116, and A549 cell lines (IC_50_ > 50 μM).

**Figure 1:**
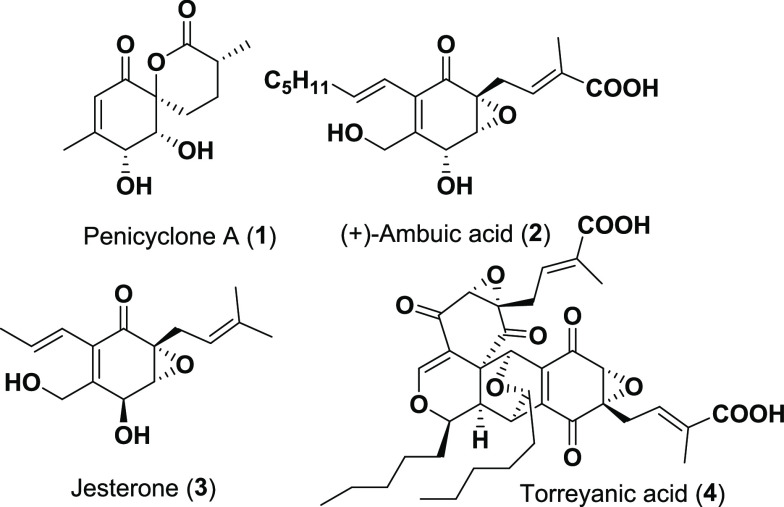
Structure of penicyclone A, ambuic acid, jestrone, and torreyanic acid.

Penicyclone A is a derivative of ambuic acid, which, along with the structurally related jesterone as well as the dimeric torreyanic acid, has been a synthetic target for some time (Figure 1).^7^ In contrast to these derivatives, penicyclone A features a unique six-membered spirolactone adjacent to a highly substituted cyclohexanone core. This variety of functional groups and chiral centers in a relatively small molecule presents a considerable synthetic challenge. Herein, we report the first total synthesis of penicyclone A, which was accomplished in 10 steps starting from a known d-ribose derivative.

## Results and Discussion

**Scheme 1:**
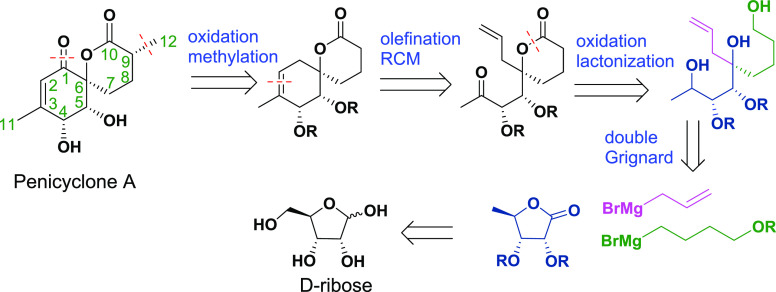
Retrosynthesis of Penicyclone A (**1**)

Our retrosynthetic analysis (Scheme 1) eventually led to d-ribose, which could be used as a cheap and optically pure source of the *cis*-diol moiety. The challenge with using a carbohydrate precursor was turning the interrupted carbon chain into the cyclohexane ring of penicyclone A.

**Scheme 2:**
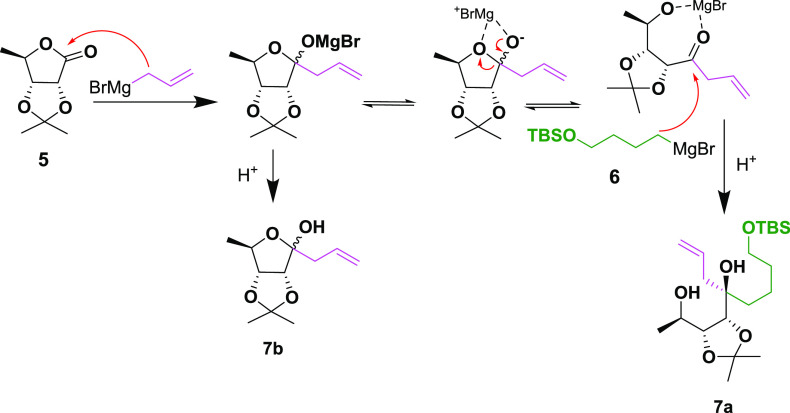
Proposed Mechanism of the Double Grignard Reaction

We envisioned that a double Grignard reaction of **5** (obtained from d-ribose in four steps with 67% overall yield) with allylmagnesium bromide and **6** would enable a diastereoselective construction of the tertiary alcohol **7a** with substituents that would later become parts of both rings. It is generally regarded that the addition of Grignard reagents to esters or lactones forms tertiary alcohols with two identical substituents owing to the higher reactivity of the ketone intermediate. However, there are several reports on specific substrates that demonstrate the possibility of a mono addition.^8^ We hypothesized that a sequential addition (1 eq of the first Grignard reagent followed by the addition of the second) might be possible when using protected sugar-derived lactones as starting materials. This transformation could lead to the diastereoselective formation of tertiary alcohols due to chelation control during the second nucleophilic attack (Scheme 2).

We thus reacted **5** at low temperature with allylmagnesium bromide followed by the addition of TBS-protected 4-hydroxybutylmagnesium bromide **6**. To our delight, the assumption was correct, and **7a** was obtained as a single diastereomer as determined by ^1^H NMR. Using this approach, **8** was obtained on the gram scale after TBAF-mediated silyl deprotection in an isolated yield of 57% over two steps from **5**. The major side product **7b** could be readily separated by column chromatography. Both the relative and absolute configuration of the TBS-protected tertiary alcohol **7a** were confirmed by X-ray diffraction. This is, to the best of our knowledge, the first example of a diastereocontrolled synthesis of tertiary alcohols from lactones using the Grignard reaction.

**Scheme 3:**
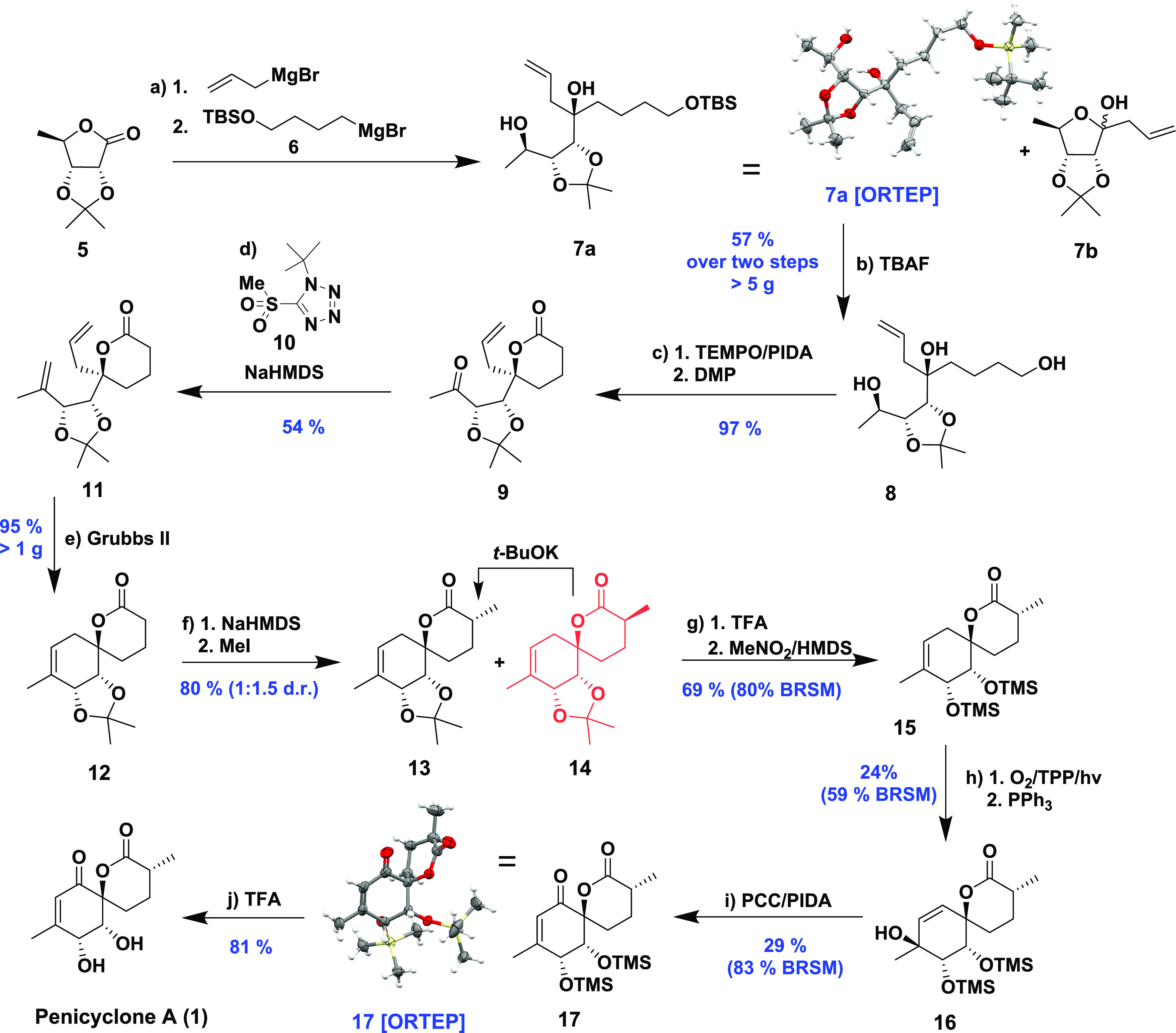
Synthetic Path to Penicyclone A Reagents and conditions: (a) 1.0 equiv AllylMgBr then 2.0 equiv TBSO(CH_2_)_4_MgBr, Et_2_O/THF, −78 °C to rt. (b) TBAF, THF/DCM, rt., 57%, over two steps. (c) TEMPO, PIDA then DMP, DCM, rt., 97%. (d) **10**, NaHMDS, THF, −78 °C to rt., 54%. (e) Grubbs II, toluene, rt., 200 mbar, 95%. (f) NaHMDS then MeI, THF, −78 °C to rt., 80%. (g) TFA, H_2_O, DCM then evaporation, HMDS, MeNO_2_, rt., 69% (80% BRSM). (h) O_2_, TPP, hν, then PPh_3_, rt., CDCl_3_, 24% (59% BRSM). (i) PCC, PIDA, DCM, rt., 29% (83% BRSM). (j) TFA, MeOH, rt., 81%. The thermal ellipsoids were drawn at a 50% probability level.

With **8** in hand, our focus was set on closing the lactone ring (Scheme 3). To that end, we first attempted reacting **8** with silver carbonate on Celite. This method is used to oxidize primary alcohols to aldehydes, which form intramolecular semiacetals (cyclization with tertiary OH group) that are quickly oxidized to lactones.^9^ After several runs, we observed significant batch-to-batch variations in reaction time and yield. We then turned to the TEMPO/PIDA^10^ catalytic system. These conditions efficiently closed the lactone ring but oxidized the secondary alcohol slowly and only partially. Thus, after complete conversion to the lactone was confirmed by TLC, Dess–Martin periodinane (DMP) was added, resulting in a one-pot formation of **9** in 97% yield on gram scale.

The methylenation of the newly installed ketone was explored next. Surprisingly, the compound proved inert to classical olefination reagents such as phosphorus yilide and titanium-based reagents. This result was rationalized to be due to steric hindrance from two neighboring rings, so we tried using smaller reagents. After extensive experimentation, we found that the diene **11** could be obtained using **10** in a modified Julia–Kociensky reaction.^11^ Subsequent ring closing metathesis proceeded smoothly to produce the advanced spirolactone intermediate **12** in an excellent 95% yield on gram scale. This intermediate closely resembles penicyclone A, requiring only the installation of the methyl group at C-9 and the carbonyl functionality at C-1. Our initial plan was to use an enantiomerically pure methylated derivative of the Grignard reagent **6**, introducing the C-9 methyl at an early stage (see the SI). Unfortunately, the Julia–Kociensky reaction with C-9-methylated **9** proceeded in low yield and resulted in complete epimerization regardless of the conditions used. This forced us to introduce the methyl group at a late stage using MeI and NaHMDS on compound **12**, yielding **13** and its C-9 epimer **14** in a 1:1.5 d.r. and 80% combined yield. After chromatographic separation of **13**, compound **14** could be epimerized to a 1:1.25 mixture of diastereomers in 92% yield using a catalytic amount of KO*t*Bu in THF to afford additional amounts of **13**.

**Scheme 4:**
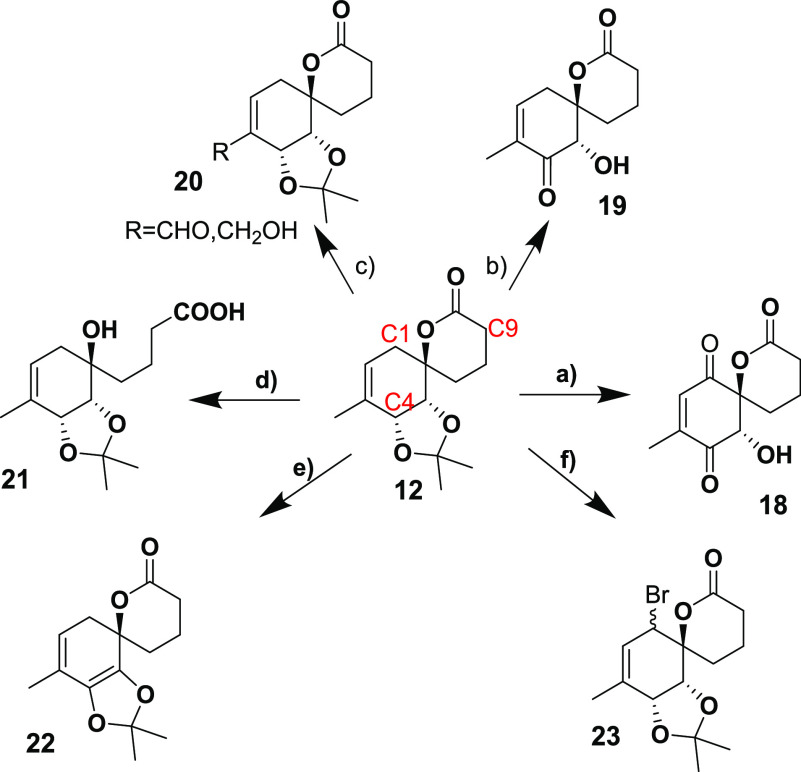
Products Obtained from the Allylic Oxidation of **12** Reagents used: (a) Rh_2_(cap)_4_(CH_3_CN)_2_/TBHP, (b) SeO_2_/KH_2_PO_4_/MeNO_2_ or NHS/Na_2_Cr_2_O_7_ or CuI/TBHP or Pd/C/TBHP, (c) SeO_2_/KH_2_PO_4_/PhMe, (d) SeO_2_/dioxane/H_2_O, (e) CrO_3_/3,5-DMP, (f) NBS.

The final challenge was the allylic oxidation at C-1. To facilitate our pursuit of the right oxidation conditions, we used **12** as a model compound since it was easier to obtain. Our initial screening focused on methods that could provide the enone directly. Oxidation of **12** using the Rh_2_(cap)_4_(CH_3_CN)_2_/TBHP system developed by Doyle and coworkers yielded **18** (Scheme 4) as the major product along with significant substrate decomposition.^12^ On the other hand, using SeO_2_/KH_2_PO_4_ in nitromethane,^13^ NHS/Na_2_Cr_2_O_7_^14^ in acetone, CuI/TBHP^15^ in acetonitrile, or Pd/C/TBHP^16^ in DCM, **19** was obtained as the major product. This indicated that hydrogen abstraction at C-4 is the dominant oxidation pathway when radical oxidants were used. Next, we explored SeO_2_-based allylic oxidation in toluene with KH_2_PO_4_ under reflux, which yielded a mixture of alcohol and aldehyde **20**. The reaction of **12** with stoichiometric SeO_2_ in dioxane with phosphate buffer resulted in ester hydrolysis yielding **21**, while the reaction in unbuffered dioxane led to complete decomposition of the starting material. This was also the result when the reaction was performed using catalytic SeO_2_ in DCM with TBHP as the stoichiometric oxidant.

Surprisingly, upon exploration of chromium-based oxidants, the substrate proved to be inert toward PCC oxidation under various conditions. Oxidation using the CrO_3_/3,5-DMP system yielded **22**, indicating once again the higher reactivity at C-4 in contrast to C-1.^17^ In order to activate the C-1 position, we also explored the allylic bromination of **12** using NBS in CCl_4_, which resulted in the formation of **23**, albeit in a moderate yield. Unfortunately, further oxidation to the enone using PNO and silver salts resulted in elimination instead.^18^

**Scheme 5:**
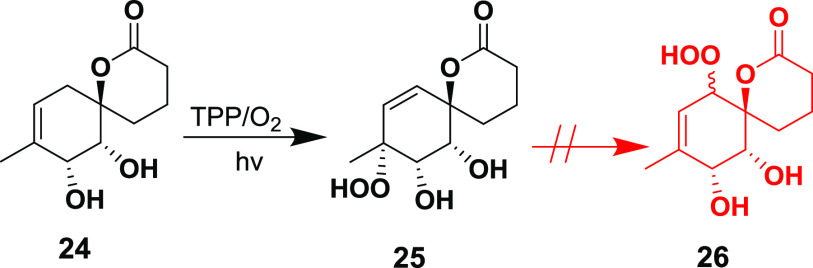
Photooxygenation of Unprotected Diol **24**

The photooxygenation of **12** was explored next, but the reaction did not occur regardless of the photosensitizer or solvent used. This lack of reactivity was rationalized by an unfavorable H—CH—C=C dihedral angle in the singlet oxygen perepoxide transition state. We hypothesized that the removal of the *cis*-diol protecting group would enable a hydroxyl group-directed singlet oxygen ene reaction on the previously sterically inaccessible face of the double bond.^19^ To this end, we removed the protecting group using TFA in DCM and obtained the unprotected diol **24**. To our delight, the oxidation of the diol **24** proceeded smoothly and delivered the peroxide **25** as a single diastereomer (Scheme 5). Our first plan relied on a Schenk rearrangement of **25** that would yield **26**, which in turn could be dehydrated to the enone.^20^ However, the rearrangement did not occur under a variety of tested conditions, likely due to strong intramolecular hydrogen bonding.

**Scheme 6:**
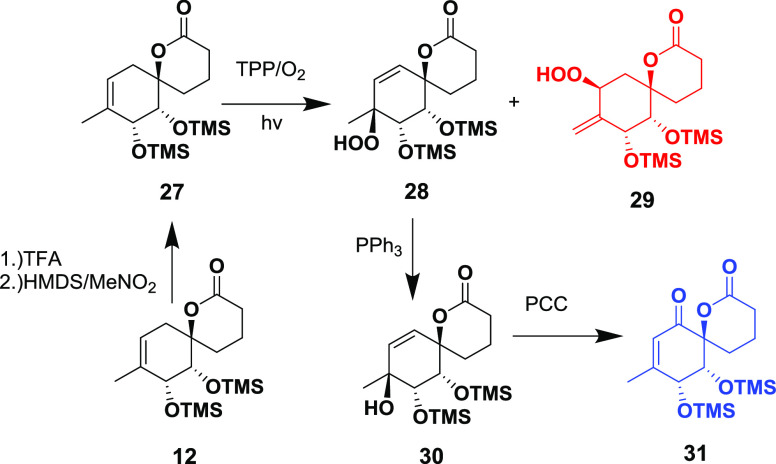
Photooxygenation of TMS-Protected Diol **27**

The other option was to reduce the peroxide to the tertiary alcohol and use an oxidative rearrangement to introduce the enone functionality. This would require the reprotection of the *cis*-diol, which could not be performed regioselectively due to the presence of the tertiary alcohol. Thus, a protecting group swap^21^ was conducted before the photooxygenation, inducing the conformational change that was required for the reaction to proceed and, on the other hand, enable further oxidative rearrangement (Scheme 6). Upon the reaction with singlet oxygen, **27** yielded a mixture of **28** and **29** due to loss of hydrogen bonding, which directed the reaction in the case of compound **24**. Finally, after reductive workup, **30** was successfully oxidized to the enone **31** using a PCC/PIDA system.^22^ It should be noted that the Schenk rearrangement of **28** was examined as well, but it produced only small amounts of the rearranged product at high temperatures, accompanied by substantial substrate decomposition.

With an end game strategy in hand, this method was used on the C-9 methylated substrate **15** yielding alcohol **16**, which was oxidatively rearranged to the TMS-protected enone **17**. Compound 17 was characterized by SCXRD, and the presence of silicium atoms in TMS enabled assignment of its absolute configuration. Removal of both TMS groups using TFA in methanol yielded the final product, penicyclone A (**1**). The spectral data of synthetic **1** (NMR, CD, and HRMS) matched the data for the originally reported sample. The final product was additionally characterized using SCXRD. The only parameter that differs from the reported natural compound is the optical rotation measured for the synthetic compound ([**α**]**_*D*_^23^** −206.0 (*c* 0.10, CHCl_3_)).

The biological activity of penicyclone A was reevaluated on the synthetic sample. The antimicrobial activity was tested against *S. aureus* (ATCC 29213), *Enterococcus faecalis* (ATCC 29212), *Moraxella catarrhalis* (ATCC 23246), and *Escherichia coli* (ECM 1556) in two separate laboratories, and the compound showed no antimicrobial activity (MIC > 32 μg/mL) on all tested strains (see the SI). The previously reported results for isolated penicyclone A could be due to a highly potent impurity present in the isolated sample.^1^

## Conclusions

In summary, we performed the first asymmetric total synthesis of penicyclone A, which was accomplished in 10 steps starting from a known ribose derivative. We chose the target molecule as it exhibited significant antimicrobial activity toward *S. aureus* after it was recently isolated from a deep-sea fungus Penicillium sp. F23-2. The key synthesis step was the construction of a tertiary alcohol using a diastereoselective double Grignard reaction on a modified sugar. We recognize that this methodology might offer a simple approach to various complex tertiary alcohols, and we are currently investigating the reaction mechanism and its scope. Another significant challenge was the late-stage introduction of the enone, which was accomplished by using a photooxygenation/oxidative rearrangement sequence. Upon reevaluation of the reported biological activity, the compound showed no antimicrobial activity against the tested bacterial strains.

## Experimental Section

All reactions were carried out under an inert argon atmosphere with dry solvents under anhydrous conditions unless otherwise stated. Volatile solvents were removed under reduced pressure rotary evaporation at 35 °C. Reactions were monitored by thin layer chromatography (TLC) carried out on 0.2 mm Merck silica plates (60F254), using UV light as the visualizing agent and KMnO_4_ and heat as the developing agent. Dichloromethane (DCM) and methanol (MeOH) were dried using activated 3 Å molecular sieves. Tetrahydrofuran (THF), diethyl ether (Et_2_O), and toluene were distilled over sodium before use. Methyl-1-(*tert*-butyl)-5-(methylsulfonyl)-1*H*-tetrazole (**10**),^11^ Dess–Martin periodinane (DMP),^23^ 5-deoxy-2,3-isopropylidene-d-ribonolactone (**5**),^24^ and (4-bromobutoxy)(*tert*-butyl)dimethylsilane (**32**)^25^ were prepared according to literature procedures. All other reagents were acquired from commercial sources and used without further purification. Yields refer to chromatographically and spectroscopically (^1^H NMR) homogeneous material unless otherwise stated. Silica gel chromatography was performed using Merck silica gel (60, particle size 0.040–0.063 mm). NMR spectra were recorded on Bruker Ascend 400 and Bruker Ascend 600 instruments at 298 K and were calibrated using residual undeuterated solvent as an internal reference (CHCl_3_: ^1^H NMR δ = 7.26 ppm, ^13^C NMR δ = 77.16 ppm). The following abbreviations were used to explain NMR peak multiplicities: s = singlet, d = doublet, t = triplet, q = quartet, m = multiplet, br = broad. High-resolution mass spectra (HRMS) were recorded on a Thermo Fisher Q extractive ESI orbitrap mass spectrometer. Circular dichroism (CD) spectra were recorded on a JASCO J815 spectrophotometer in methanol (spectroscopic grade) using appropriate 1 cm path quartz cuvettes. Optical rotations were measured on a Schmidt Haensch Polartronic NH8 polarimetar using a 10 cm cuvette.

### Synthesis of Compound **6**


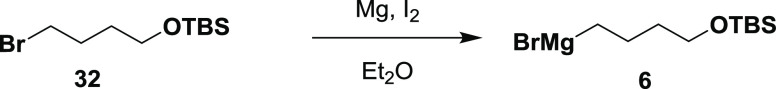
To a suspension of magnesium turnings (1.2 g, 49.4 mmol, 1.06 equiv) in Et_2_O (20 mL) was added I_2_ (1 mg) then **32** (12.4 g, 46.4 mmol, 1 equiv) was added over 5 min. During the addition, the reaction mixture started refluxing without external heating and the reflux was maintained by external heating (oil bath) after the addition was complete for 1 h. The reaction mixture was cooled to room temperature and immediately used in the next step.

### Synthesis of Compound **7a**


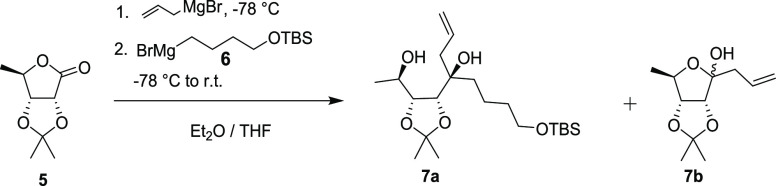
To a solution of lactone **5** (4.00 g, 23.2 mmol, 1.0 equiv) in Et_2_O (150 mL) and THF (23 mL) at −78 °C, a solution of allyl magnesium bromide (23.2 mL, 1.0 M in Et_2_O, 1.0 equiv) was added dropwise over 40 min. The reaction was stirred for 35 min at −78 °C, and then, a freshly prepared solution of **6** (2.0 equiv) was added over 10 min. The resulting suspension was warmed to room temperature over 45 min, stirred at room temperature for 30 min, and then quenched by the addition of sat. NH_4_Cl (80 mL). After stirring for 10 min, H_2_O (10 mL) was added, and the layers were separated. The aqueous layer was extracted twice with Et_2_O (50 mL), and the combined organic extracts were washed with brine (30 mL), dried with Na_2_SO_4_, filtered, and concentrated. The crude product was purified by column chromatography (15% EtOAc/Hex to 30% EtOAc/Hex) to give **7a** as a single diastereomer containing **7b** (ca. 15 mol %, according to ^1^H NMR), which was taken into the next step without further purification (6.525 g). An analytically pure sample of **7a** was obtained as a white solid by a second chromatographic purification (50% EtOAc / DCM).

Compound **7a**:

Rf = 0.48 (30% EtOAc/Hex, KMnO_4_); [**α**]**_*D*_^23^** +22.9 (*c* 1.05, CHCl_3_);

IR (ATR) ν_max_ (cm^–1^): 3270, 2978, 2952, 2930, 2858, 1638, 1099, 773;

HRMS (ESI-Orbitrap) *m*/*z*: [M + Na]^+^ calcd for C_21_H_42_O_5_SiNa 425.2699; found 425.2704;

^1^H NMR (600 MHz, CDCl_3_): δ/ppm 5.84–5.77 (m, 1H), 5.19–5.14 (m, 2H), 4.30 (bs, 1H), 3.98 (d, 1H, *J* = 4.74 Hz), 3.99–3.94 (m, 1H), 3.76 (dd, *J* = 9.7 Hz, 4.7 Hz, 1H), 3.65–3.59 (m, 2H), 2.68 (bs, 1H), 2.49 (dq, *J* = 14.2 Hz, 7.6 Hz, 2H), 1.82–1.76 (m, 1H), 1.67–1.60 (m, 1H), 1.58–1.42 (m, 4H), 1.43 (s, 3H), 1.32 (s, 3H), 1.26 (d, *J* = 6.3 Hz, 3H), 0.89 (s, 9H), 0.05 (s, 6H);

^13^C{^1^H} NMR (151 MHz, CDCl_3_): δ/ppm 133.3, 119.6, 107.1, 82.0, 80.8, 74.7, 65.3, 63.0, 41.5, 35.0, 33.2, 28.3, 26.1, 26.0, 19.9, 19.2, 18.5, −5.1.

### Synthesis of Compound **8**


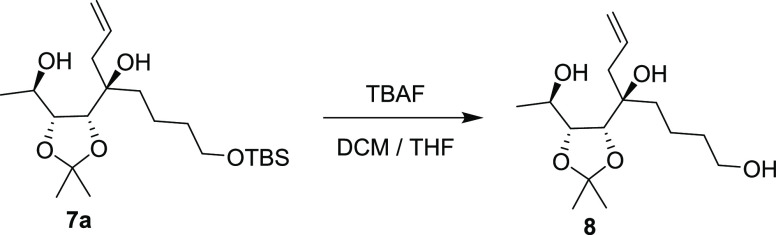
To a solution of **7a** (6.525 g, 16 mmol) in DCM (42 mL) was added TBAF (42 mL, 1.0 M in THF), and the resulting orange solution was stirred at room temperature for 16 h. The reaction mixture was concentrated and purified by column chromatography (EtOAc) to give **8** (3.87 g, 57% over two steps) as a viscous, colorless oil.

Compound **8**:

Rf = 0.36 (EtOAc, KMnO_4_); [**α**]**_*D*_^23^** +35.1 (*c* 0.74, CHCl_3_);

IR (ATR) ν_max_ (cm^–1^): 3306, 2984, 2935, 2872, 1640, 1051;

HRMS (ESI-Orbitrap) *m*/*z*: [M + Na]^+^ calcd for C_15_H_28_O_5_Na 311.1834; found 311.1835;

^1^H NMR (400 MHz, CDCl_3_): δ/ppm 5.85–5.74 (m, 1H), 5.20–5.13 (m, 2H), 4.58 (bs, 1H), 4.00–3.93 (m, 1H), 3.97 (d, J = 4.7, 1H), 3.76 (dd, *J* = 9.7 Hz, 4.7 Hz, 1H), 3.65 (t, *J* = 5.9 Hz, 2H), 3.30 (bs, 1H), 2.49 (d, *J* = 7.5 Hz, 2H), 2.11 (bs, 1H), 1.84–1.76 (m, 1H), 1.67–1.41 (m, 5H), 1.43 (s, 3H), 1.32 (s, 3H), 1.27 (d, *J* = 6.2 Hz, 3H);

^13^C{^1^H} NMR (100 MHz, CDCl_3_): δ/ppm 133.3, 119.4, 107.1, 81.8, 80.8, 74.4, 65.4, 62.5, 41.3, 34.8, 32.9, 28.3, 25.9, 19.9, 18.9.

### Synthesis of Compound **9**


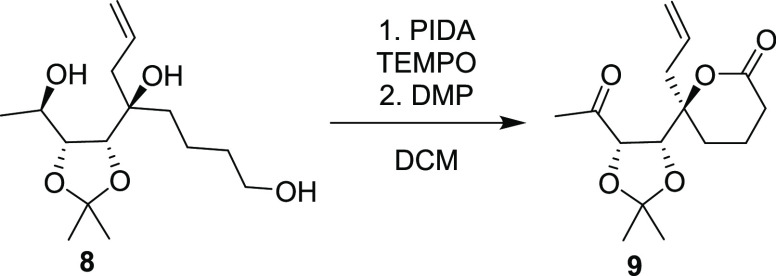
To a solution of **8** (150 mg, 0.52 mmol, 1 equiv) in DCM (5 mL) open to air was added PIDA (840 mg, 2.61 mmol, 5 equiv) and TEMPO (16.5 mg, 0.106 mmol, 20 mol %). The mixture was stirred at room temperature for 90 min, DMP (310 mg, 0.73 mmol, 1.4 equiv) was added, and the mixture was stirred for an additional 2 h and 10 min. The reaction was quenched by dilution with Et_2_O (10 mL), the resulting suspension was concentrated, and the crude residue was purified by column chromatography (30% EtOAc/Hex), giving **9** (143 mg, 97%) as a pale yellow oil that crystallized overnight to a white solid.

Compound **9**:

Rf = 0.50 (30% EtOAc/Hex, KMnO_4_); [**α**]**_*D*_^23^** −23.0 (*c* 0.89, CHCl_3_);

IR (ATR) ν_max_ (cm^–1^): 3086, 2978, 1731, 1703, 1645;

HRMS (ESI-Orbitrap) *m*/*z*: [M + H]^+^ calcd for C_15_H_23_O_5_ 283.1545; found 283.1545;

^1^H NMR (600 MHz, CDCl_3_): δ/ppm 5.76–5.65 (m, 1H), 5.24–5.17 (m, 2H), 4.41 (d, *J* = 7.9 Hz, 1H), 4.35 (d, *J* = 7.9 Hz, 1H), 2.88–2.82 (m, 1H), 2.53–2.31 (m, 3H), 2.27 (s, 3H), 2.07–2.00 (m, 1H), 1.95–1.74 (m, 3H), 1.68 (s, 3H), 1.38 (s, 3H);

^13^C{^1^H} NMR (151 MHz, CDCl_3_): δ/ppm 212.2, 169.7, 132.0, 120.5, 109.8, 84.0, 82.3, 80.8, 40.3, 29.5, 28.9, 26.6, 24.5, 23.7, 16.0.

### Synthesis of Compound **11**


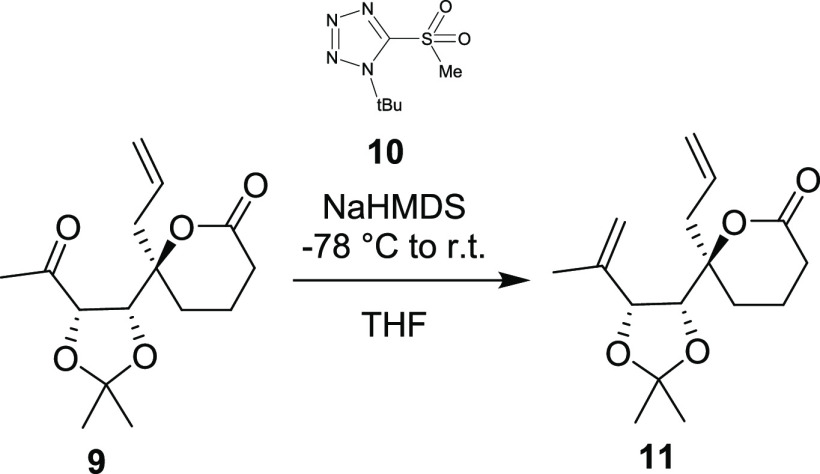
To a solution of **9** (2.63 g, 9.32 mmol, 1 equiv) and **10** (2.81 g, 18.65 mmol, 2 equiv) in THF (130 mL) at −78 °C was added NaHMDS (12.14 mL, 12.14 mmol, 1.0 M in THF, 1.3 equiv) at once, and the solution was left to slowly warm to room temperature overnight (16 h). The reaction was quenched with saturated aqueous NH_4_Cl (40 mL), the layers were separated, and the aqueous layer was extracted with Et_2_O (2 × 50 mL). The combined organic extracts were dried over Na_2_SO_4_ and concentrated. The crude residue was purified by column chromatography (DCM to 20% EtOAc/DCM), giving **11** (1.40 g, 54%) as a white, crystalline solid.

Compound **11**:

Rf = 0.63 (15% EtOAc/DCM, KMnO_4_); [**α**]**_*D*_^23^** −24.2 (*c* 1.1, CHCl_3_);

IR (ATR) ν_max_ (cm^–1^): 3079, 2982, 2963, 2913, 1728, 1642, 1035;

HRMS (ESI-Orbitrap) *m*/*z*: [M + Na]^+^ calcd for C_16_H_24_O_4_Na 303.1572; found 303.1569;

^1^H NMR (400 MHz, CDCl_3_): δ/ppm 5.79 (m, 1H), 5.19–5.13 (m, 2H), 5.08 (m, 1H), 5.02 (m, 1H), 4.65 (d, *J* = 6.8 Hz, 1H), 4.22 (d, *J* = 6.8 Hz, 1H), 2.60 (dq, *J* = 15.4 Hz, 7.4 Hz, 2H), 2.50–2.43 (m, 1H), 2.40–2.31 (m, 1H), 1.96–1.73 (m, 7H), 1.59 (s, 3H), 1.37 (s, 3H);

^13^C{^1^H} NMR (100 MHz, CDCl_3_): δ/ppm 170.5, 143.2, 132.4, 119.9, 114.7, 108.0, 85.0, 80.7, 80.3, 40.5, 30.0, 26.5, 25.5, 24.9, 20.9, 16.6.

### Synthesis of Compound **12**


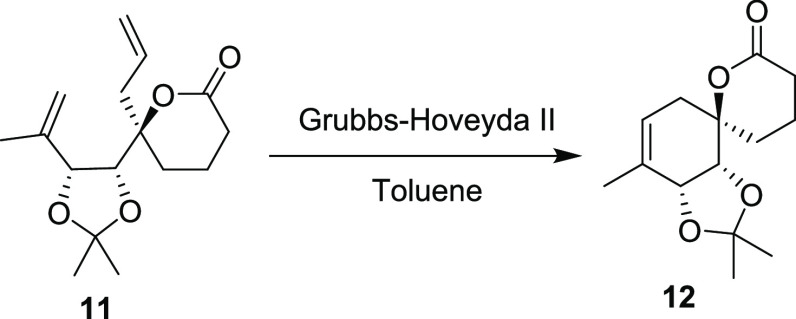
To a solution of **11** (1.40 g, 5 mmol, 1 equiv) in toluene (125 mL) was added Grubbs-Hoveyda II (100 mg, 0.16 mmol, 3 mol %) catalyst, and the mixture was stirred at room temperature and 200 mBar. After 3 h, the mixture was concentrated, and the crude residue was purified by column chromatography (60% EtOAc/Hex), giving **12** (1.20 g, 95%) as an off-white solid due to traces of ruthenium. This compound could be further purified by an additional column chromatography (60% EtOAc/Hex), but this impurity did not affect the next steps and got removed during the next purification.

Compound **12**:

Rf = 0.46 (60% EtOAc/Hex, KMnO_4_); [**α**]**_*D*_^23^** −73.6 (*c* 1.0, CHCl_3_);

IR (ATR) ν_max_ (cm^–1^): 2964, 2913, 1731, 1044;

HRMS (ESI-Orbitrap) *m*/*z*: [M + Na]^+^ calcd for C_14_H_20_O_4_Na 275.1259; found 275.1257;

^1^H NMR (600 MHz, CDCl_3_): δ/ppm 5.33 (m, 1H), 4.53 (d, *J* = 5.5 Hz, 1H), 4.15 (dd, *J* = 5.5 Hz, 1.0 Hz, 1H), 2.59–2.48 (m, 2H), 2.40–2.35 (m, 1H), 2.27–2.22 (m, 1H), 2.08–2.03 (m, 1H), 2.00–1.93 (m, 1H), 1.91–1.84 (m, 1H), 1.83–1.79 (m, 1H), 1.78 (m, 3H), 1.39 (s, 3H), 1.36 (s, 3H);

^13^C{^1^H} NMR (151 MHz, CDCl_3_): δ/ppm 170.6, 132.9, 118.8, 109.8, 83.2, 76.6, 76.3, 33.5, 30.0, 29.8, 27.5, 26.9, 19.5, 16.0.

### Synthesis of Compound **13**


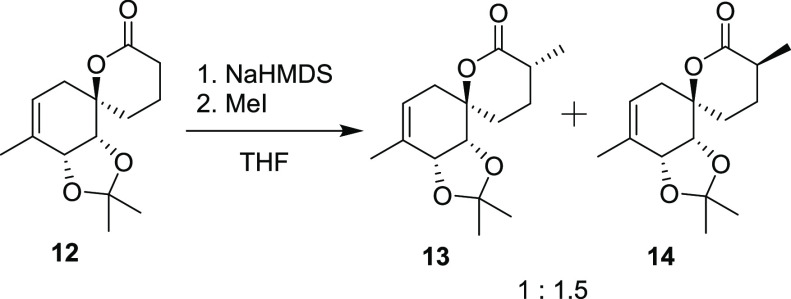
To a solution of **12** (200 mg, 0.793 mmol, 1 equiv) in THF (6.5 mL) at −78 °C was added NaHMDS (800 μL, 1 M in THF, 1.01 equiv) dropwise over 5 min. The mixture was stirred at −78 °C for 50 min, and methyl iodide (100 μL, 1.6 mmol, 2 equiv) was added dropwise. The mixture was warmed to −50 °C over 2 h and then warmed to room temperature over 30 min. The mixture was concentrated and purified by column chromatography (30% EtOAc/Hex) to give **13** and **14** (170.4 mg, 80%) as a 1:1.5 mixture of diastereomers. The diastereomers could be separated by column chromatography (10% EtOAc/Hex), and both were obtained as white crystalline solids.

Compound **13**:

Rf = 0.33 (30% EtOAc/Hex, KMnO_4_); [**α**]**_*D*_^23^** −85.3 (*c* 0.40, CHCl_3_).

IR (ATR) ν_max_ (cm^–1^): 2987, 2918, 1723, 1028;

HRMS (ESI-Orbitrap) *m*/*z*: [M + H]^+^ calcd for C_15_H_23_O_4_ 267.1591; found 267.1595;

^1^H NMR (600 MHz, CDCl_3_): δ/ppm 5.35–5.33 (m, 1H), 4.54 (d, *J* = 5.5 Hz, 1H), 4.12 (dd, *J* = 5.7 Hz, 0.7 Hz, 1H), 2.56–2.50 (m, 1H), 2.36–2.24 (m, 2H), 2.05–1.96 (m, 3H), 1.78 (m, 3H), 1.68–1.61 (m, 1H), 1.39 (s, 3H), 1.37 (s, 3H), 1.30 (d, *J* = 7.1 Hz, 3H);

^13^C{^1^H} NMR (151 MHz, CDCl_3_): δ/ppm 174.1, 133.0, 119.1, 109.7, 83.4, 78.2, 76.4, 35.7, 33.3, 29.8, 27.4, 26.8, 24.8, 19.6, 17.8.

Compound **14**:

Rf = 0.39 (30% EtOAc/Hex, KMnO_4_);

HRMS (ESI-Orbitrap) *m*/*z*: [M + H]^+^ calcd for C_15_H_23_O_4_ 267.1591; found 267.1583;

^1^H NMR (600 MHz, CDCl_3_): δ/ppm 5.32–5.31 (m, 1H), 4.52 (d, *J* = 5.2 Hz, 1H), 4.15 (dd, *J* = 5.4 Hz, 1.0 Hz, 1H), 2.49–2.42 (m, 1H), 2.42–2.37 (m, 1H), 2.27–2.18 (m, 2H), 1.96–1.91 (m, 1H), 1.80–1.69 (m, 5H), 1.39 (s, 3H), 1.35 (s, 3H), 1.31 (d, J = 7.1 Hz, 3H);

^13^C{^1^H} NMR (151 MHz, CDCl_3_): δ/ppm 174.1, 132.7, 119.0, 109.8, 83.3, 76.2, 75.6, 35.6, 34.5, 30.1, 27.6, 27.1, 24.6, 19.5, 17.5.

### Epimerization of Compound **14**


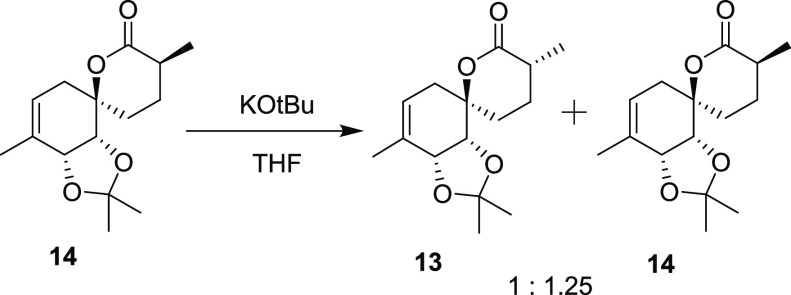
To a solution of **14** (104 mg, 0.39 mmol, 1 equiv) in THF (1.9 mL) was added KO*t*Bu (1.9 mL, 0.01 M solution in THF, 5 mol %), and the mixture was stirred at room temperature for 10 min. The reaction was quenched with ammonium chloride (15 mg) and stirred for 5 min. The mixture was filtered, and the solids were washed twice with Et_2_O (3 mL) and concentrated. The crude residue was purified by column chromatography (30% EtOAc/Hex) to give **13** and **14** (95.4 mg, 92%) as a 1:1.25 mixture of diastereomers. The diastereomers could be separated by column chromatography (10% EtOAc/Hex).

### Synthesis of Compound **15**


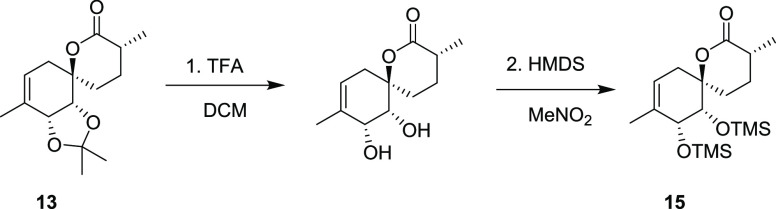
To a solution of **13** (51 mg, 0.192 mmol, 1 equiv) in DCM (1 mL) open to air was added TFA (1 mL) and H_2_O (25 μL). The mixture was stirred at room temperature for 1 h and 30 min. The mixture was concentrated, the crude residue was dissolved in MeNO_2_ (1 mL), and HMDS (100 μL, 0.477 mmol, 2.48 equiv) was added. The mixture was stirred at room temperature for 5 min and concentrated, and the crude residue was purified by column chromatography (Hex to 10% EtOAc/Hex), giving **15** (49 mg, 0.132 mmol, 69%, 80% BRSM) as a colorless oil and recovered starting material **13** (7.3 mg, 0.0274 mmol, 14%).

Compound **15**:

Rf = 0.61 (30% EtOAc/Hex, KMnO_4_); [**α**]**_*D*_^23^** −119.4 (*c* 1.0, CHCl_3_);

IR (ATR) ν_max_ (cm^–1^): 2956, 2878, 1731, 834;

HRMS (ESI-Orbitrap) *m*/*z*: [M + Na]^+^ calcd for C_18_H_34_O_4_Si_2_Na 393.1893; found 393.1893;

^1^H NMR (600 MHz, CDCl_3_): δ/ppm 5.25–5.23 (m, 1H), 4.09 (d, J = 3.5 Hz, 1H), 3.79 (d, J = 4.1 Hz, 1H), 2.49–2.41 (m, 1H), 2.40–2.31 (m, 1H), 2.28–2.19 (m, 1H), 2.13–2.03 (m, 1H), 1.94–1.86 (m, 2H), 1.71–1.69 (m, 3H), 1.57–1.46 (m, 1H), 1.26 (d, J = 7.1 Hz, 3H), 0.15 (s, 9H), 0.15 (s, 9H);

^13^C{^1^H} NMR (151 MHz, CDCl_3_): δ/ppm 174.9, 135.0, 119.5, 85.7, 75.6, 73.0, 36.9, 36.3, 27.0, 25.4, 20.5, 17.4, 0.8, 0.7.

### Synthesis of Compound **16**


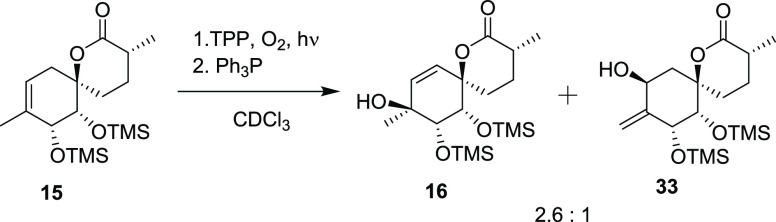
To a solution of **15** (49 mg, 132 μmol, 1 equiv) in CDCl_3_ (5 mL) under an atmosphere of O_2_ was added TPP (2 mg, 3.25 μmol, 2.5 mol %), and the solution was irradiated with a 250 W tungsten halogen lamp projector at room temperature. After 14 h, Ph_3_P (45 mg, 172 μmol, 1.3 equiv) was added, and the mixture was stirred for an additional 5 min. The mixture was concentrated, and the crude residue was purified by column chromatography (25% EtOAc/Hex), giving **16** (12.38 mg, 32 μmol, 24%, 59% BRSM) as a colorless oil, **33** (4.72 mg, 9%, 23% BRSM) as a colorless oil, and recovered starting material **15** (29.0 mg, 78 μmol, 59%).

Compound **16**:

Rf = 0.26 (25% EtOAc/Hex, KMnO_4_); [**α**]**_*D*_^23^** −87.1 (*c* 0.51, CHCl_3_);

IR (ATR) ν_max_ (cm^–1^): 3426, 2957, 1727, 837;

HRMS (ESI-Orbitrap) *m*/*z*: [M + Na]^+^ calcd for C_18_H_34_O_5_Si_2_Na 409.1842; found 409.1844;

^1^H NMR (400 MHz, CDCl_3_): δ/ppm 5.75 (bs, 1H), 5.60 (bs, 1H), 4.25 (bs, 1H), 3.74 (bs, 1H), 2.42–2.17 (m, 2H), 1.97–1.85 (m, 2H), 1.70–1.58 (m, 2H), 1.29–1.27 (m, 6H), 0.16 (s, 9H), 0.14 (s, 9H);

^13^C NMR: Due to peak broadening caused by intramolecular hydrogen bonding, a ^13^C NMR spectrum could not be acquired even with prolonged acquisition times. The ^1^H NMR peaks sharpened at higher temperatures, though this was accompanied by decomposition.

### Synthesis of Compound **17**


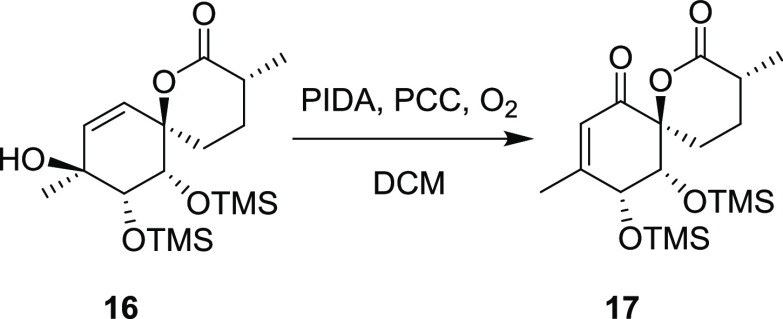
To a solution of **16** (15.0 mg, 38.8 μmol, 1 equiv) in DCM (300 μL) under an atmosphere of O_2_ was added PIDA (37.1 mg, 114.9 μmol, 3 equiv) and PCC (1.0 mg, 4.7 μmol, 14 mol %). The mixture was stirred at room temperature for 18 h and then directly purified by column chromatography (25% EtOAc/Hex), giving **17** (4.33 mg, 11.3 μmol, 29%, 83% BRSM) as a white, crystalline material and recovered starting material **16** (9.72 mg, 65%).

Compound **17**:

Rf = 0.33 (25% EtOAc/Hex, UV, and KMnO_4_); [**α**]**_*D*_^23^** −197.4 (*c* 0.23, CHCl_3_);

IR (ATR) ν_max_ (cm^–1^): 2952, 1741, 1677, 1643, 836;

HRMS (ESI-Orbitrap) *m*/*z*: [M + H]^+^ calcd for C_18_H_33_O_5_Si_2_ 385.1867; found 385.1866;

^1^H NMR (600 MHz, CDCl_3_): δ/ppm 5.88 (m, 1H), 4.19 (bd, *J* = 3.50 Hz, 1H), 4.02 (d, *J* = 4.06 Hz, 1H), 2.41–2.31 (m, 1H), 2.26–2.11 (m, 2H), 2.02 (d, *J* = 1.31 Hz, 3H), 1.81–1.74 (m, 1H), 1.58–1.45 (m, 1H), 1.31 (d, *J* = 6.90 Hz, 3H), 0.20 (s, 9H), 0.18 (s, 9H);

^13^C{^1^H} NMR (151 MHz, CDCl_3_): δ/ppm 195.7, 174.2, 159.4, 124.8, 73.7, 72.9, 35.9, 31.1, 25.13, 25.10, 22.1, 17.0, 0.78, 0.72.

### Synthesis of Compound **1**


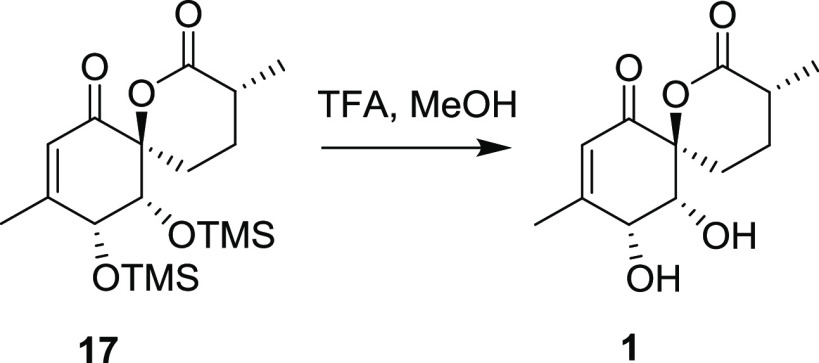
To a solution of **10** (10.36 mg, 26.94 μmol, 1 equiv) in MeOH (1 mL) was added TFA (10 μL), and the mixture was stirred at room temperature open to air for 20 min. The mixture was concentrated, and the crude residue was purified by column chromatography (EtOAc), giving **1** (5.25 mg, 21.83 μmol, 81%) as a colorless crystalline material.

Compound **1**:

Rf = 0.33 (25% EtOAc/Hex, UV, and KMnO_4_); [**α**]**_*D*_^23^** -206.0 (*c* 0.10, CHCl_3_);

IR (ATR) ν_max_ (cm^–1^): 3452, 2943, 2878, 1721, 1665, 1639, 1227;

HRMS (ESI-Orbitrap) *m*/*z*: [M + Na]^+^ calcd for C_12_H_16_O_5_Na 263.0895; found 263.0894;

^1^H NMR (600 MHz, DMSO-*d*_6_): δ/ppm 5.86 (d, J = 1.4 Hz, 1H), 5.67 (bs, 1H), 5.60 (bs, 1H), 4.16 (bs, 1H), 3.83 (bs, 1H), 2.43–2.36 (m, 1H), 2.21 (td, *J* = 14.4 Hz, 4.0 Hz, 1H), 2.01 (d, *J* = 1.3 Hz, 3H), 1.99 (dt, *J* = 15.1 Hz, 3.6 Hz, 1H), 1.78–1.73 (m, 1H), 1.27–1.19 (m, 1H), 1.11 (d, *J* = 6.9 Hz, 3H);

^13^C{^1^H} NMR (151 MHz, DMSO-*d*_6_): δ/ppm 196.1, 173.8, 162.0, 123.4, 87.8, 70.8, 69.3, 34.7, 24.6, 21.6, 16.9.

## Supporting Information Available

NMR characterization of substrates, optimization of reaction conditions, comparative tables of the spectroscopic data of the natural and the synthetic products, X-ray crystallographic data, summary of oxidation studies, in vitro antibacterial assay, and references (PDF)
