# Supplementary material for: Total Synthesis of Penicyclone A Using a Double Grignard Reaction
Source: J Org Chem. 2022 Nov 16;87(23):16054–62. doi: 10.1021/acs.joc.2c02200 (PMC9724088; doi:10.1021/acs.joc.2c02200)

Total synthesis of Penicyclone A using a double Grignard reaction

Supporting information

Gregor Talajić, Edi Topić, Jerko Meštrović, Nikola Cindro*

ncindro.chem@pmf.hr

Department of Chemistry, Faculty of Science, University of Zagreb, Horvatovac 102a, 10000 Zagreb, Croatia

Contents

Skeletal numbering of Penicyclone A S-1
Initial optimization of the double grignard addition S-2
Summary of the allylic oxidation studies on **12** S-3
Synthesis of the 9-(R)-methyl analogue of **9** S-4
Penicyclone A spectral comparison S-5
X-ray crystal structures S-7
CD spectrum of Penicyclone A S-19
In vitro antibacterial assay of Penicyclone A S-20
References S-21
NMR spectra S-23

Skeletal numbering of Penicyclone A

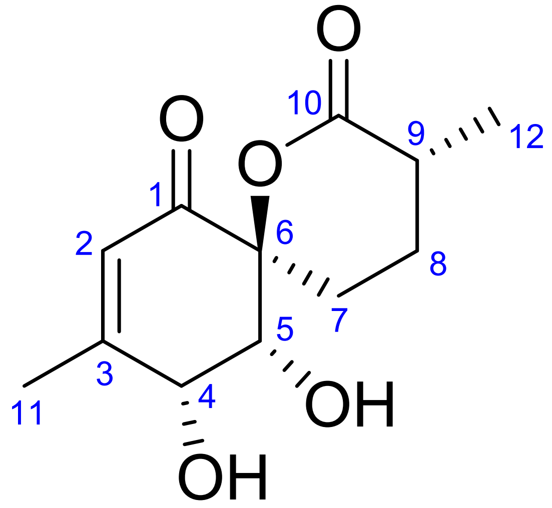

Initial optimization of the double Grignard addition

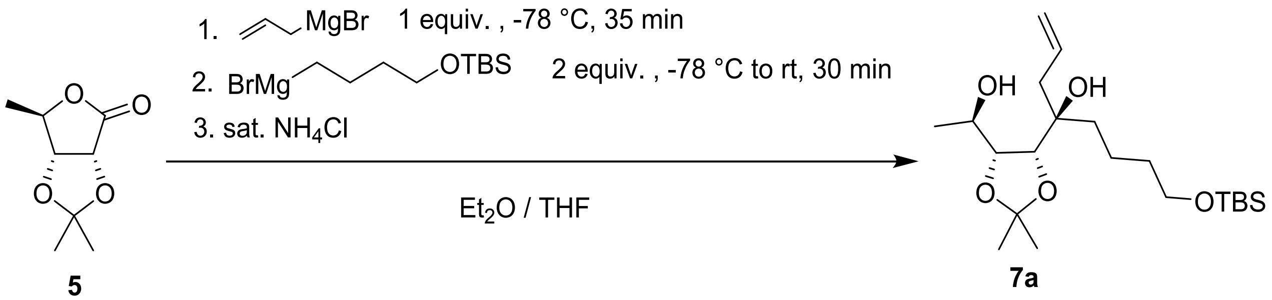

Variation from conditions presented above	Yield of 7a after TBS deprotection
No variation	57%
2h instead of 35 min before 2nd addition	55%
1h at rt instead of 30 min before quench	57%
16h at rt instead of 30 min before quench	decomposition
THF instead of Et_2O : THF (5 : 1)	46%

Summary of the allylic oxidation studies on **12**

Conditions	Result
**SeO_2 Dioxane**	Decomposition
**SeO_2 (cat.) TBHP DCM**	Decomposition
**PCC, DCM**	No reaction
**PCC, DMSO**	No reaction
**K_2CO_3 (0.5 equiv), $Rh_2(cap)_4(ACN)_2$ (2 mol%), TBHP (5 equiv), DCM, 24h, rt**	A (24%) + B (11%) + rsm (64%)
**SeO_2 (2.2 equiv), KH_2PO_4 (3 equiv) Toluene, 2h, 110 °C**	C (46%%) + D (8%)
**SeO_2 (2.2 equiv), KH2PO4 (3 equiv), Nitromethane, 10 min, 60 °C**	E (25%) + rsm (71%)
**SeO_2 KH_2PO_4, Dioxane : H_2O (9:1), 21h, 60°C**	Lactone hydrolysis
**CuI (1 mol%), TBHP (3 equiv), DCM / ACN (1:1), 16h, rt**	E (from crude NMR), decomposition
**NHS, $Na_2Cr_2O_7$, Acetone, 16h, rt**	E (27%) + rsm (62%)
**O_2, NHPI, Bz_2O_2, EtOAc / Acetone (1:1), 20h, 55 °C**	E (from crude NMR)
**NHPI, PCC, Acetone**	No reaction
**Pd/C, TBHP K_2CO_3, DCM, 16h, rt**	E (17%) + rsm (79%)
**CrO_3 3,5-DMP, DCM, 2h, -25 °C**	B (12%) + rsm (21%)
**NBS, hν, CCl_4, 25 min, rt**	F (61%)
**Singlet oxygen**	No reaction
**Cr(V) (7 equiv), DCM, 6h, rt**	B (from crude NMR), decomposition

Cr(v) = sodium bis(2-ethyl-2-hydroxybutanoato)$^{2-}$oxochromate(V)

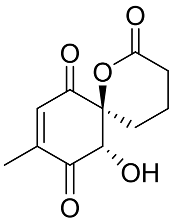

A

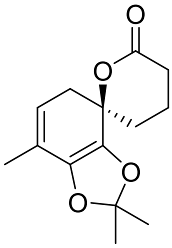

B

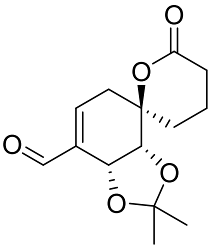

C

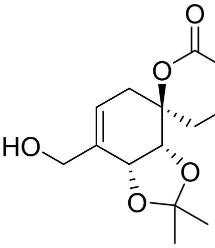

D

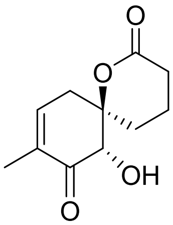

E

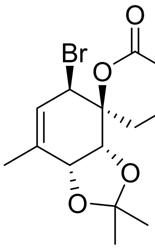

F

Synthesis of the 9-(R)-methyl analogue of **9**

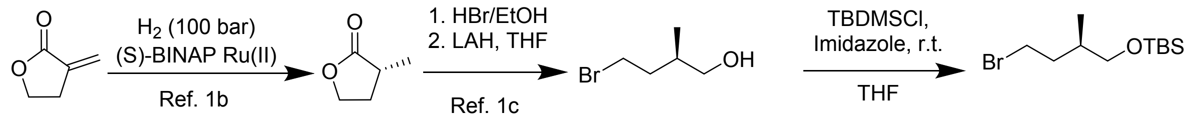

(R)-4-bromo-2-methylbutan-1-ol obtained by literature procedures[1] was used instead of 4-bromobutan-1-ol in the synthetic sequence under the conditions described for the desmethyl derivative without detectable C-9 epimerization in any of the synthetic steps. Unfortunately, the modified Julia Kociensky methylenation proceeded in low yield with complete C-9 epimerization.

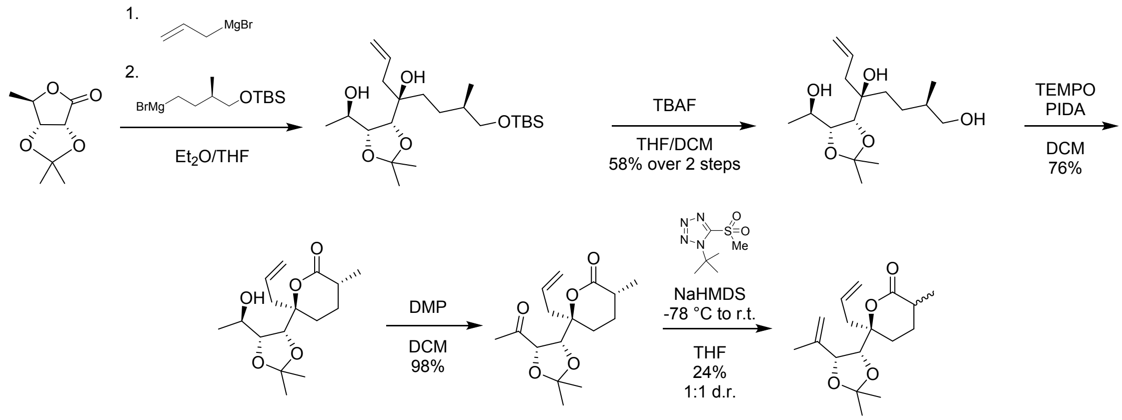

Penicyclone A spectral comparison

Position	^{1}H NMR<br>Natural sample<br>(500 MHz in DMSO)	^{1}H NMR<br>Synthetic sample<br>(600 MHz in DMSO)
1		
2	5.86 (s, 1H)	5.86 (d, J = 1.4 Hz, 1H)
3		
4	4.15 (dd, J = 5.7 Hz, 4.6 Hz, 1H)	4.16 (bs, 1H)
5	3.82 (dd, J = 6.3 Hz, 4.6 Hz, 1H)	3.83 (bs, 1H)
6		
7	2.21 (dt, J = 14.4 Hz, 3.2 Hz, 1H)<br>2.00 (dt J = 14.4 Hz, 3.2 Hz, 1H)	2.21 (td, J = 14.4 Hz, 4.0 Hz, 1H)<br>1.99 (dt, J = 15.1 Hz, 3.6 Hz, 1H)
8	1.75 (m, 1H)<br>1.24 (m, 1H)	1.78 – 1.73 (m, 1H)<br>1.27 – 1.19 (m, 1H)
9	2.38 (m, 1H)	2.43 – 2.36 (m, 1H)
10		
11	2.01 (s, 3H)	2.01 (d, J= 1.3 Hz, 3H)
12	1.11 (d, J = 6.9 Hz, 3H)	1.11 (d, J = 6.9 Hz, 3H)
OH - 4	5.68 (d, J = 5.7 Hz, 1H)	5.67 (bs, 1H)
OH - 5	5.61 (d, J= 6.3 Hz, 1H)	5.60 (bs, 1H)

Position	^{13}C NMR<br>Natural sample<br>(500 MHz in DMSO)	^{13}C NMR<br>Synthetic sample<br>(600 MHz in DMSO)
1	196.0	196.1
2	123.4	123.4
3	162.0	162.0
4	69.3	69.3
5	70.9	70.8
6	87.8	87.8
7	24.6	24.6
8	24.6	24.6
9	34.7	34.7
10	173.8	173.8
11	21.6	21.6
12	16.9	16.9

Comparison of ^{1}H and $^{13}C\{^{1}H\}$ NMR spectra of natural and synthetic Penicyclone A in MeOD.

Blue spectrum corresponds to natural Penicyclone A while the red spectrum corresponds to a synthetic sample.

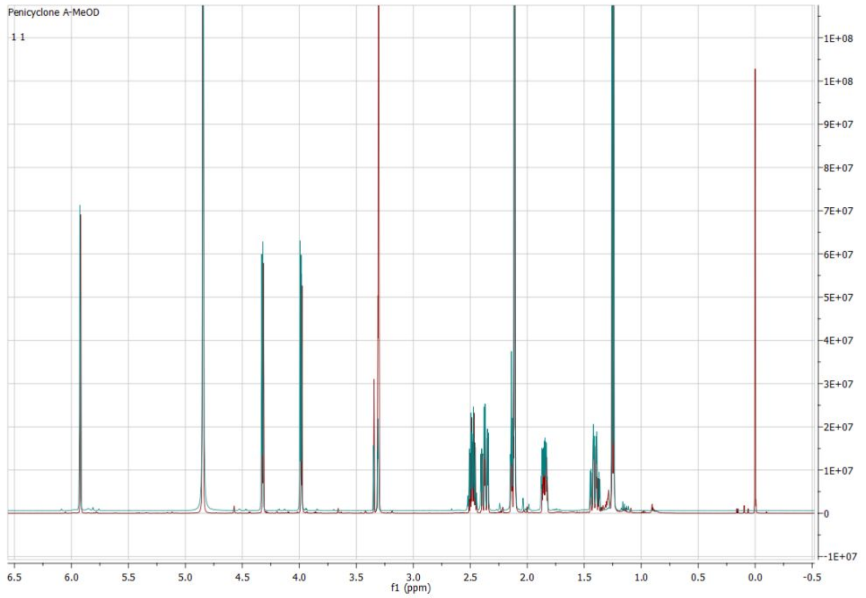

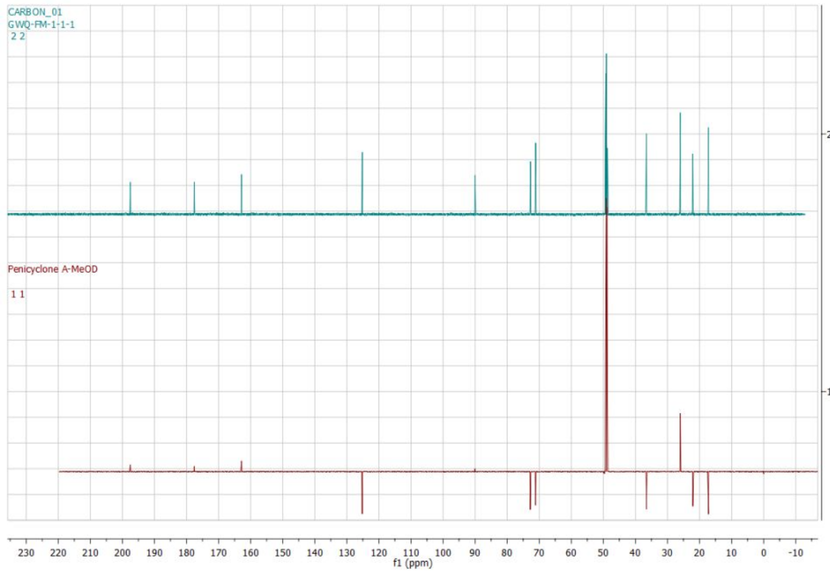

X-ray crystal structures

High-quality single crystals of **7a**, **17** and **1** were prepared by recrystallization of purified compounds in hexane (**7a**), diethyl ether (**17**) and ethyl acetate (**1**). Diffracted intensities were collected on Rigaku XtaLAB Synergy diffractometer equipped with Dualflex source and HyPix detector, using ω-scans. Data were prepared using the CrysAlis program package.[2] A summary of general and crystal data, intensity data collection and final refinement parameters are presented in Table S1. The structures were solved with dual space methods using SHELXT.[3] The refinement procedure by full-matrix least-squares methods based on F^2 values against all reflections included anisotropic displacement parameters for all non-H atoms. Hydrogen atoms bound to carbon atoms were placed in geometrically idealized positions and refined by the use of the riding model with $U_{iso} = 1.2U_{eq}$ of the connected carbon atom or as ideal CH_3 groups with $U_{iso} = 1.5U_{eq}$. Hydrogen atoms attached to heteroatoms were located in the difference Fourier maps at the final stages of the refinement procedure. Their coordinates were refined freely, with $U_{iso} = 1.2U_{eq}$ of the parent atom. All refinements were performed using SHELXL-2013.[4] The SHELX programs operated within the Olex2 suite.[5] Geometrical calculations and molecular graphics were done with Mercury.[6] The thermal ellipsoids were drawn at a 50% probability level.

Compound **7a** crystallizes in the chiral space group $P2_1$ with two symmetrically inequivalent molecules in the asymmetric unit (Figures S1, S2). All stereocenters (residue 1: C9, C10, C14, C15; residue 2: C36, C37, C41, C42) have *R* configuration. Relevant bond lengths and angles for both residues are shown in Tables S1 and S3.

Compound **17** crystallizes in the chiral space group $P2_12_12_1$ with one molecule in the asymmetric unit (Figures S3, S4). Stereocentres C2, C3, and C10 have *R* configuration, while stereocenter C1 has *S* configuration. Relevant bond lengths and angles are shown in Table S4.

Compound **1** crystallizes in the chiral space group $P2_1$ with two molecules in the asymmetric unit (Figures S5, S6). Crystal structure, molecular structure and stereochemistry of the compound is identical to the reported Penicyclone A (CSD REFCODE: RUVSOS).[7] Relevant bond lengths and angles are shown in Table S5.

Table S1. General and crystal data, a summary of intensity data collection and structure refinement for compounds **7a**, **17** and **1**

Identification code	**7a**	**17**	**1**
Empirical formula	$C_{21}H_{42}O_5Si$	$C_{18}H_{32}O_5Si_2$	$C_{12}H_{16}O_5$
M_r	402.63	384.61	240.25
T/K	169.98(10)	169.99(10)	169.99(10)
Crystal system	monoclinic	orthorhombic	monoclinic
Space group	$P2_1$	$P2_12_12_1$	$P2_1$
a/Å	8.07760(10)	9.50840(10)	9.25140(10)
b/Å	10.88140(10)	12.2840(2)	7.37840(10)
c/Å	28.7346(4)	18.8828(3)	17.0057(2)

α/°	90	90	90
β/°	97.4920(10)	90	102.0930(10)
γ/°	90	90	90
V/Å^3	2504.08(5)	2205.53(6)	1135.06(2)
Z	4	4	4
ρ_{calc}/g cm^{-3}	1.068	1.158	1.406
μ/mm^{-1}	1.023	1.65	0.920
F(000)	888	832	512
Crystal size/mm^3	0.472 × 0.127 × 0.067	0.08 × 0.05 × 0.03	0.172 × 0.086 × 0.081
Radiation	Cu Kα (λ = 1.54184 Å)		
2Θ range/°	6.204 to 159.766	8.588 to 155.452	5.314 to 156.354
Index ranges	$-10 \leq h \leq 10$<br>$-13 \leq k \leq 13$<br>$-36 \leq l \leq 36$	$-12 \leq h \leq 11$,<br>$-15 \leq k \leq 15$,<br>$-23 \leq l \leq 22$	$-11 \leq h \leq 10$,<br>$-9 \leq k \leq 9$,<br>$-21 \leq l \leq 21$
Reflections collected	16412	23787	27992
Independent reflections	16412 [Merged R_{int}, R_{sigma} = 0.0197]	4679 [R_{int} = 0.0509, R_{sigma} = 0.0369]	4784 [R_{int} = 0.0361, R_{sigma} = 0.0255]
Data/restraints/ parameters	16412/1/506	4679/0/234	4784/5/332
g_1, g_2 in w[a]	0.1652, 0.9259	0.0423, 0.3379	0.036, 0.156
Goodness–of–fit on F^2, S[b]	1.042	1.033	1.088
Final R and wR[c] values [$I \geq 2\sigma(I)$]	R_1 = 0.0705, wR_2 = 0.2047	R_1 = 0.0344, wR_2 = 0.0820	R_1 = 0.0275, wR_2 = 0.0714
Final R and wR[c] values [all data]	R_1 = 0.0714, wR_2 = 0.2076	R_1 = 0.0379, wR_2 = 0.0834	R_1 = 0.0280, wR_2 = 0.0718
Largest diff. peak/hole / e Å^{-3}	0.727/–0.585	0.20/–0.18	0.18/–0.16
Flack parameter, x [d]	0.04(5)	0.002(13)	0.04(5)

[a] $w = 1/[\sigma^2(F_o^2) + (g_1P)^2 + g_2P]$ where $P = (F_o^2 + 2F_c^2)/3$

[b] $S = \{\Sigma[w(F_o^2 - F_c^2)^2]/(N_r - N_p)\}^{1/2}$ where N_r = number of independent reflections, N_p = number of refined parameters.

[c] $R = \Sigma||F_o| - |F_c||/\Sigma|F_o|$; $wR = \{\Sigma[w(F_o^2 - F_c^2)^2]/\Sigma[w(F_o^2)^2]\}^{1/2}$

[d] $I(hkl) = (1 - x)|F(hkl)|^2 + x|F(-h-k-l)|^2$, where x is the refined Flack parameter.

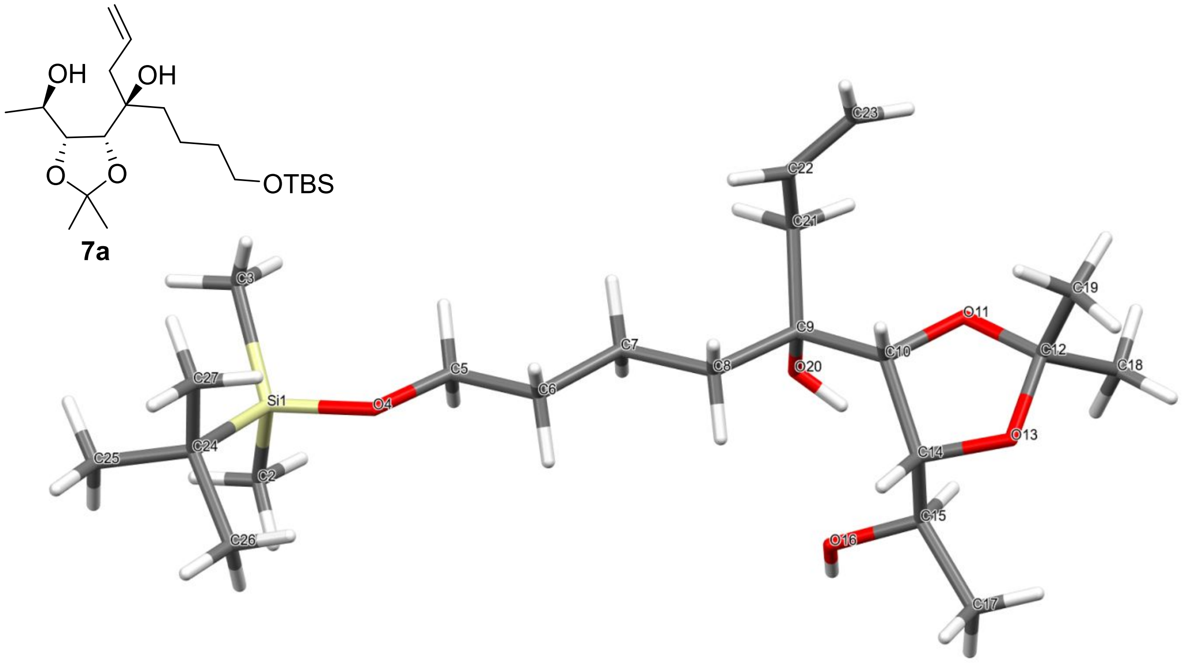

7a

(a)

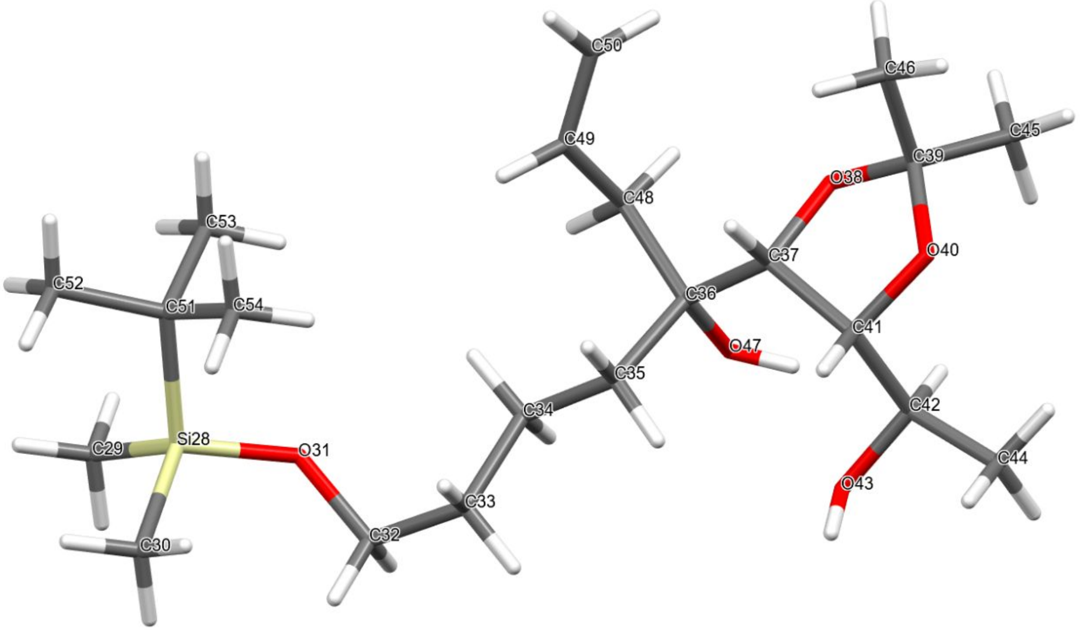

(b)

Figure S1. Atom enumeration in first (a) and second (b) symmetrically inequivalent molecules in the crystal structure of compound **7a**.

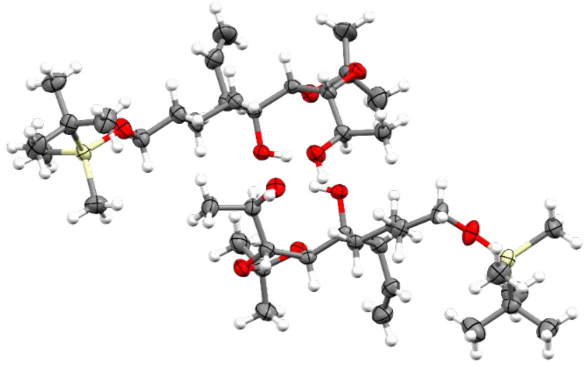

(a)

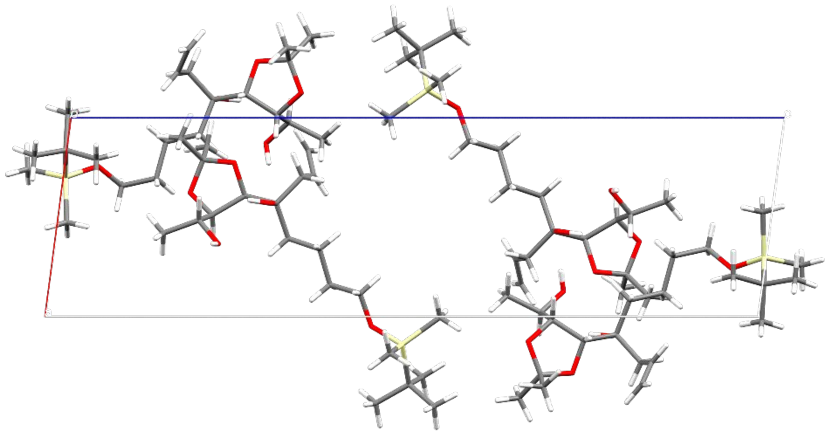

(b)

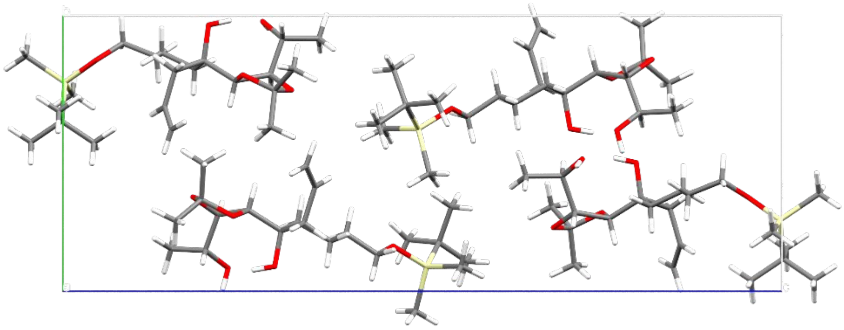

(c)

Figure S2. (a) Asymmetric unit, (b) crystal packing along the *a*-axis and (c) crystal packing along the *b*-axis for the crystal structure of compound **7a**.

Table S2. Bond lengths and angles between non-hydrogen atoms in the first residue for the crystal structure of compound **7a**.

Bond	Length/Å	Bonds	Angle/°	Bonds	Angle/°
C5–C6	1.506(10)	O4–C5–C6	110.5(6)	Si1–C24–C25	109.8(6)
C6–C7	1.527(10)	C5–C6–C7	110.2(6)	C25–C24–C26	109.1(7)
C7–C8	1.523(9)	C6–C7–C8	113.7(6)	Si1–C24–C26	109.2(6)
C8–C9	1.526(9)	C7–C8–C9	114.6(6)	C25–C24–C27	109.3(8)
C9–C10	1.535(8)	O20–C9–C21	108.0(5)	Si1–C24–C27	110.1(5)
C9–C21	1.538(10)	C10–C9–C21	108.6(5)	C26–C24–C27	109.4(8)
C10–C14	1.536(8)	O20–C9–C8	106.1(5)	Si1–O4–C5	122.4(4)
C12–C18	1.515(13)	C8–C9–C10	109.2(5)	C10–O11–C12	105.3(5)
C12–C19	1.531(13)	O20–C9–C10	112.1(5)	C12–O13–C14	108.3(5)
C14–C15	1.525(9)	C8–C9–C21	112.9(5)	O4–Si1–C2	110.4(3)
C15–C17	1.520(9)	C9–C10–C14	123.0(5)	C2–Si1–C3	109.2(5)
C21–C22	1.506(12)	O11–C10–C9	110.4(5)	O4–Si1–C3	110.3(4)
C22–C23	1.315(18)	O11–C10–C14	101.0(5)	C2–Si1–C24	110.6(4)
C24–C25	1.525(13)	O11–C12–C19	111.6(7)	O4–Si1–C24	104.2(3)
C24–C26	1.542(12)	C18–C12–C19	111.5(8)	C3–Si1–C24	112.1(4)
C24–C27	1.541(13)	O11–C12–O13	106.4(6)		
O4–C5	1.422(9)	O13–C12–C18	111.4(7)		
O11–C10	1.426(8)	O11–C12–C18	108.6(7)		
O11–C12	1.418(10)	O13–C12–C19	107.3(7)		
O13–C12	1.431(9)	C10–C14–C15	118.8(5)		
O13–C14	1.439(8)	O13–C14–C10	99.3(5)		
O16–C15	1.422(8)	O13–C14–C15	108.6(5)		
O20–C9	1.434(8)	C14–C15–C17	110.1(6)		
Si1–C2	1.864(9)	O16–C15–C14	110.6(5)		
Si1–C24	1.889(8)	O16–C15–C17	108.3(5)		
Si1–C3	1.877(9)	C9–C21–C22	114.9(7)		
Si1–O4	1.654(5)	C21–C22–C23	124.6(10)		

Table S3. Bond lengths and angles between non-hydrogen atoms in the second residue for the crystal structure of compound **7a**.

Bond	Length/Å	Bonds	Angle/°	Bonds	Angle/°
C32–C33	1.514(10)	O31–C32–C33	110.2(6)	Si28–C51–C52	110.7(6)
C33–C34	1.530(9)	C32–C33–C34	113.3(5)	C52–C51–C53	108.9(8)
C34–C35	1.520(9)	C33–C34–C35	112.1(5)	Si28–C51–C53	110.2(6)
C35–C36	1.534(9)	C34–C35–C36	114.5(5)	C52–C51–C54	109.6(8)
C36–C48	1.550(9)	O47–C36–C48	108.3(5)	Si28–C51–C54	108.8(6)
C36–C37	1.532(8)	C37–C36–C48	108.5(5)	C53–C51–C54	108.6(8)
C37–C41	1.533(8)	O47–C36–C35	106.1(5)	Si28–O31–C32	130.2(5)
C39–C45	1.515(12)	C35–C36–C37	109.4(5)	C37–O38–C39	106.2(5)
C39–C46	1.530(12)	O47–C36–C37	112.8(5)	C39–O40–C41	109.6(5)
C41–C42	1.543(10)	C35–C36–C48	111.9(5)	O31–Si28–C29	107.7(4)
C42–C44	1.513(11)	C36–C37–C41	123.3(5)	C29–Si28–C30	109.3(5)
C48–C49	1.498(11)	O38–C37–C36	109.2(5)	O31–Si28–C30	111.8(4)
C49–C50	1.329(13)	O38–C37–C41	101.6(5)	C29–Si28–C51	111.0(4)
C51–C52	1.524(14)	O38–C39–C46	111.2(6)	O31–Si28–C51	106.1(3)
C51–C53	1.538(12)	C45–C39–C46	112.1(7)	C30–Si28–C51	110.9(4)
C51–C54	1.541(14)	O38–C39–O40	105.2(5)		
O31–C32	1.412(10)	O40–C39–C45	111.7(6)		
O38–C37	1.420(8)	O38–C39–C45	108.4(6)		
O38–C39	1.417(8)	O40–C39–C46	108.0(6)		
O40–C41	1.440(9)	C37–C41–C42	119.6(5)		
O40–C39	1.435(9)	O40–C41–C37	100.0(5)		
O43–C42	1.419(9)	O40–C41–C42	107.9(5)		
O47–C36	1.429(8)	C41–C42–C44	110.4(6)		
Si28–O31	1.642(6)	O43–C42–C41	111.4(5)		
Si28–C29	1.868(9)	O43–C42–C44	108.0(6)		
Si28–C30	1.878(9)	C36–C48–C49	115.4(6)		
Si28–C51	1.883(8)	C48–C49–C50	124.3(8)		

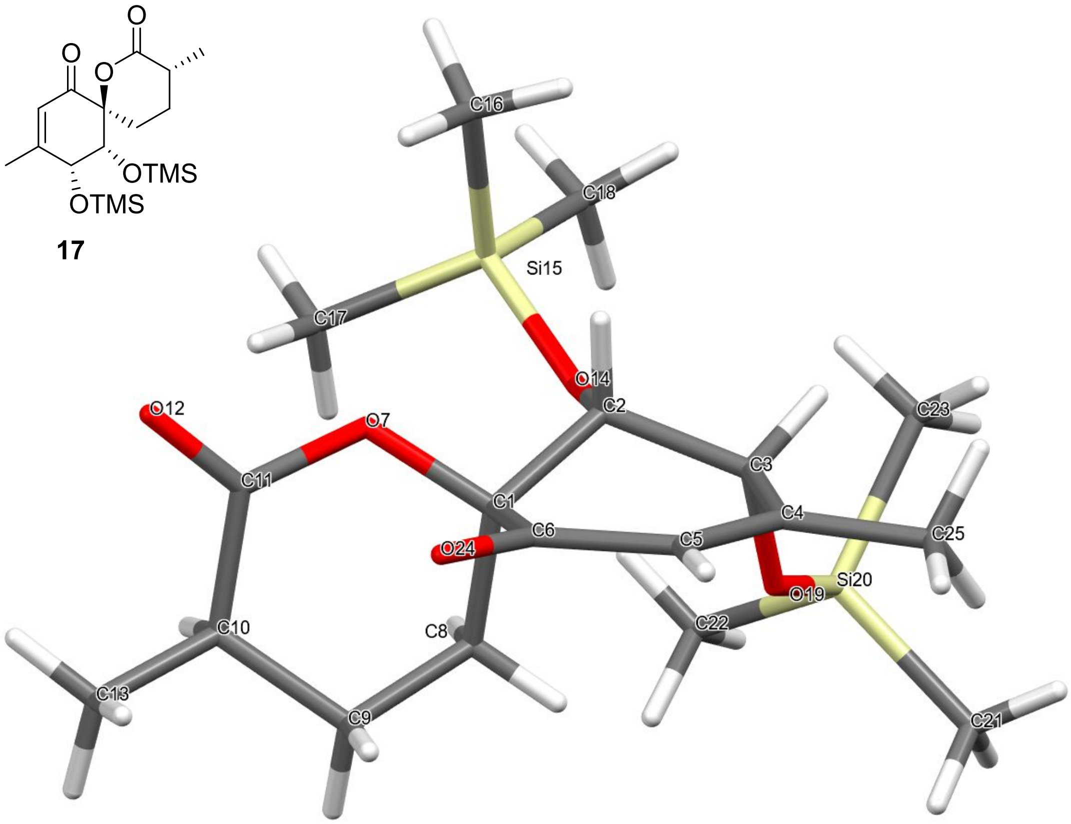

Figure S3. Atom enumeration in the crystal structure of the compound **17**.

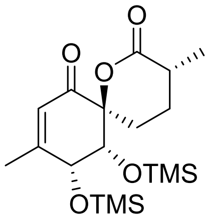

17

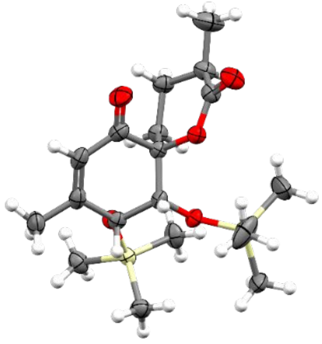

(a)

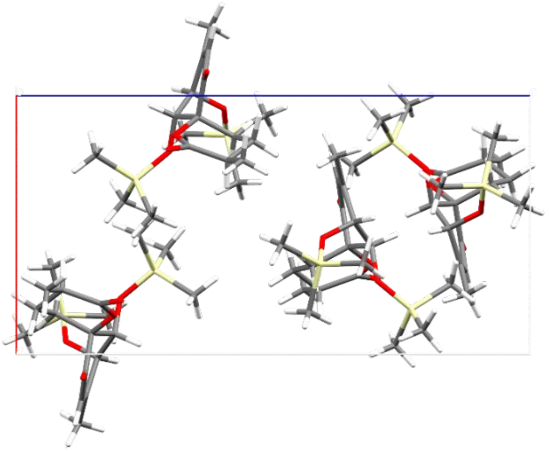

(b)

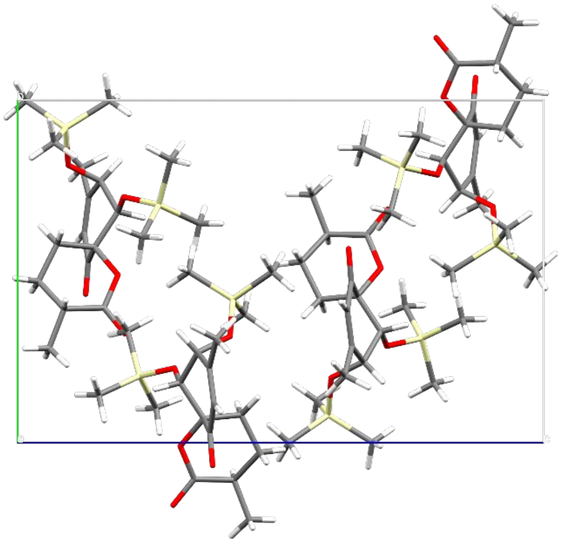

(c)

Figure S4. (a) Asymmetric unit, (b) crystal packing along the *a*-axis and (c) crystal packing along the *b*-axis for the crystal structure of the compound **17**.

Table S4. Bond lengths and angles between non-hydrogen atoms in the crystal structure of the compound **17**.

Bond	Length/Å	Bonds	Angle/°	Bonds	Angle/°
C1–C8	1.524(3)	O7–C1–C2	105.09(17)	O12–C11–C10	123.1(2)
C1–C2	1.536(3)	C2–C1–C6	109.15(17)	C1–O7–C11	121.35(18)
C1–C6	1.536(3)	O7–C1–C6	106.35(19)	Si15–O14–C2	128.56(15)
C2–C3	1.526(3)	C2–C1–C8	114.84(19)	Si20–O19–C3	128.27(16)
C3–C4	1.518(4)	O7–C1–C8	111.15(18)	O14–Si15–C16	110.51(15)
C4–C25	1.497(4)	C6–C1–C8	109.86(19)	C16–Si15–C17	112.6(2)
C4–C5	1.333(3)	O14–C2–C1	111.87(17)	O14–Si15–C17	108.36(18)
C5–C6	1.458(4)	O14–C2–C3	108.6(2)	C16–Si15–C18	109.96(19)
C8–C9	1.519(3)	C1–C2–C3	112.27(18)	O14–Si15–C18	106.76(13)
C9–C10	1.529(4)	O19–C3–C2	113.0(2)	C17–Si15–C18	108.50(17)
C10–C11	1.513(4)	O19–C3–C4	106.73(19)	O19–Si20–C21	103.92(11)
C10–C13	1.518(4)	C2–C3–C4	111.9(2)	C21–Si20–C22	109.88(13)
O7–C1	1.451(3)	C3–C4–C5	121.5(2)	O19–Si20–C22	109.93(13)
O7–C11	1.357(3)	C3–C4–C25	115.8(2)	C21–Si20–C23	110.89(12)
O12–C11	1.202(3)	C5–C4–C25	122.7(3)	O19–Si20–C23	111.19(11)
O14–C2	1.406(3)	C4–C5–C6	123.9(3)	C22–Si20–C23	110.83(13)
O19–C3	1.424(3)	C1–C6–C5	117.1(2)		
O24–C6	1.211(3)	O24–C6–C1	119.9(2)		
Si15–O14	1.6571(18)	O24–C6–C5	123.0(3)		
Si15–C16	1.849(4)	C1–C8–C9	111.1(2)		
Si15–C17	1.853(5)	C8–C9–C10	110.6(2)		
Si15–C18	1.844(4)	C9–C10–C11	113.6(2)		
Si20–O19	1.6548(19)	C9–C10–C13	112.6(3)		
Si20–C21	1.870(3)	C11–C10–C13	110.9(2)		
Si20–C22	1.865(3)	O7–C11–O12	118.0(2)		
Si20–C23	1.860(3)	O7–C11–C10	118.3(2)		

Figure S5. Atom enumeration in the crystal structure of the compound **1** (Penicyclone A).

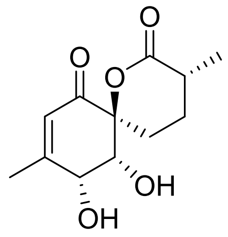

1

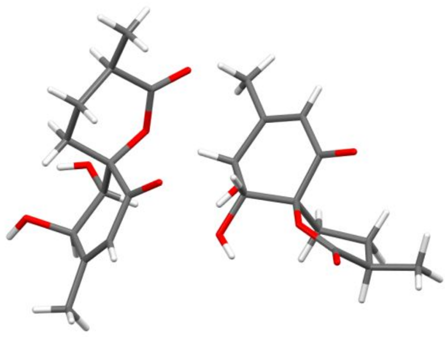

(a)

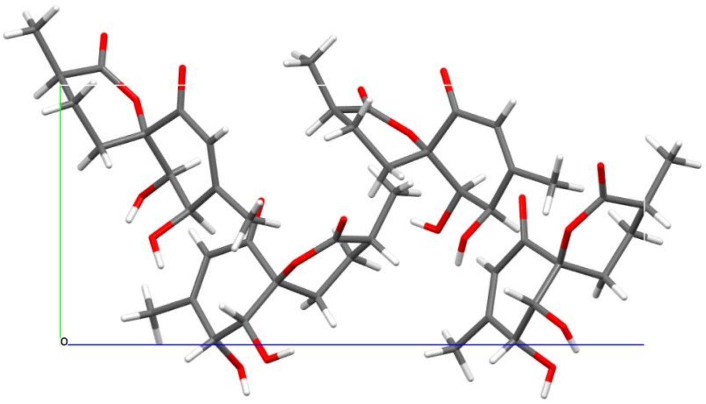

(b)

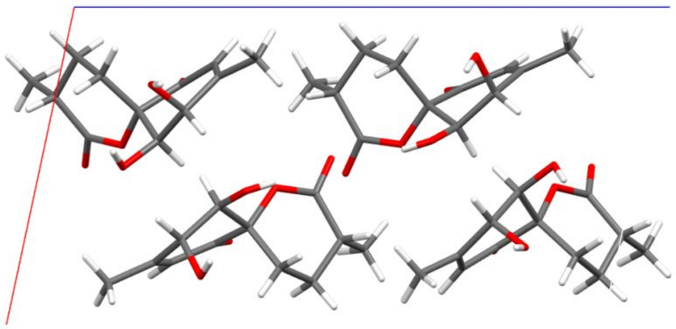

(c)

Figure S6. (a) Asymmetric unit, (b) crystal packing along the *a*-axis and (c) crystal packing along the *b*-axis for the crystal structure of the compound **1** (Penicyclone A).

Table S5. Bond lengths and angles between non-hydrogen atoms in the crystal structure of the compound **1** (Penicyclone A).

Bond	Length/Å	Bonds	Angle/°	Bonds	Angle/°
C2–C1	1.493(3)	C1–C2–C14	115.90(17)	O12–C7–C8	124.10(18)
C2–C14	1.508(3)	C2–C14–C13	113.33(15)	O12–C7–O6	116.60(17)
C2–C3	1.339(3)	C2–C3–C4	122.42(17)	O15–C14–C13	108.63(15)
C4–C3	1.462(3)	C3–C2–C1	122.41(18)	O15–C14–C2	109.24(14)
C4–C5	1.531(3)	C3–C2–C14	121.69(17)	O16–C13–C14	111.40(15)
C5–C10	1.527(2)	C3–C4–C5	117.19(16)	O16–C13–C5	111.99(15)
C5–C13	1.539(2)	C4–C5–C13	107.98(14)	O17–C4–C3	121.30(18)
C7–C8	1.513(3)	C7–C8–C11	110.93(17)	O17–C4–C5	121.49(17)
C8–C11	1.523(3)	C7–C8–C9	112.01(15)	O23–C22–C21	104.42(12)
C8–C9	1.523(3)	C7–O6–C5	124.54(14)	O23–C22–C27	111.28(13)
C9–C10	1.524(3)	C8–C9–C10	109.89(15)	O23–C22–C30	106.74(13)
C13–C14	1.522(2)	C9–C10–C5	110.07(15)	O23–C24–C25	120.81(15)
C19–C18	1.494(2)	C10–C5–C13	115.05(15)	O29–C24–C25	122.09(16)
C19–C31	1.334(3)	C10–C5–C4	109.59(14)	O29–C24–O23	116.88(15)
C20–C19	1.512(2)	C11–C8–C9	113.57(16)	O32–C30–C22	119.76(16)
C20–C21	1.533(2)	C14–C13–C5	111.99(14)	O32–C30–C31	123.22(17)
C22–C21	1.522(2)	C18–C19–C20	115.37(16)	O33–C21–C20	107.34(14)
C22–C27	1.528(2)	C19–C20–C21	111.82(14)	O33–C21–C22	113.87(14)
C22–C30	1.535(2)	C19–C31–C30	123.25(17)	O34–C20–C19	107.82(13)
C24–C25	1.515(2)	C21–C22–C27	114.36(14)	O34–C20–C21	113.02(14)
C25–C26	1.528(2)	C21–C22–C30	109.53(14)		
C25–C28	1.524(3)	C22–C21–C20	112.46(13)		
C27–C26	1.520(2)	C24–C25–C26	114.83(14)		
C30–C31	1.462(3)	C24–C25–C28	110.45(16)		
O6–C5	1.448(2)	C24–O23–C22	122.96(13)		
O6–C7	1.351(2)	C26–C27–C22	110.36(15)		
O12–C7	1.204(2)	C27–C22–C30	110.12(14)		
O15–C14	1.433(2)	C27–C26–C25	110.91(14)		

O16–C13	1.408(2)	C28–C25–C26	111.67(15)		
O17–C4	1.223(2)	C31–C19–C18	122.37(17)		
O23–C22	1.4535(19)	C31–C19–C20	122.23(16)		
O23–C24	1.345(2)	C31–C30–C22	116.98(15)		
O29–C24	1.208(2)	O6–C5–C10	111.99(14)		
O32–C30	1.220(2)	O6–C5–C13	104.68(13)		
O33–C21	1.409(2)	O6–C5–C4	107.14(15)		
O34–C20	1.429(2)	O6–C7–C8	119.12(16)		

CD spectrum of Penicyclone A

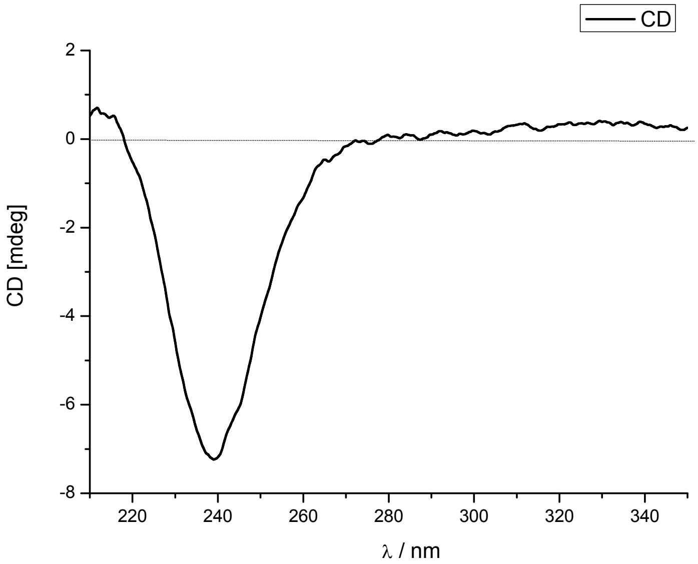

Figure S4. CD spectra of Penicyclone A recorded in methanol; c (Penicyclone A)= $2x10^{-5}$ mol dm^{-3}

In vitro antibacterial assay of Penicyclone A

Minimum inhibitory concentrations (MICs) were determined by the broth microdilution method according to guidelines of the Clinical Laboratory Standards Institute. Double dilutions of tested compounds in 96-well microtitre plates were prepared in the 128-0.5 mg/mL concentration range. E.coli and S. aureus were grown on Mueller-Hinton agar plates (by Becton Dickinson, USA) while E. faecalis and M.catarrhalis were grown on Mueller-Hinton agar with 5% sheep blood. Inoculates were prepared by direct colony suspension method and plates were inoculated with $5x10^4$ CFU/well. Results were determined by visual inspection after 20-22h incubation at 37°C in ambient air.

Bacterial strains and chemicals

Species	strain	
Staphylococcus aureus	ATCC 29213	
Enterococcus faecalis	ATCC29212	
Moraxella catarrhalis	ATCC 23246	
Escherichia coli	ECM1556	TolC-*Tn10*

Chemical	Manufac.	Cat.no.	Lot
BBL Mueller Hinton II Broth (cation adjusted)	BD	212322	4044343
azithromycin	USP	1046056	HOC212

	MIC (µg/ml)			
**Compound #**	***S. aureus* *ATCC 29213***	***E. faecalis* *ATCC29212***	***M. catarrhalis* *ATCC 23246***	***E. coli* *TolC-Tn10***
Penicyclone A	>32	>32	>32	>32
Penicillin G	0.25	4	<=0.06	32
Penicillin V	0.125	2	<=0.06	16
azithromycin	1	16	<=0.06	0.5

Results from the additional antimicrobial testing of Penicyclone A performed according to CLSI guideline.

09.06.2022. MIC results of testing from 08.06.2022.

The MIC values were read after 22 hours of incubation at 37°C.

**Test strain**	**CLSI range for Vancomycin**	**Vancomycin USP standard (composite) R107F0 (493 mg/vial → 5mg/mL)**	**Penicyclone A, Institut Ruđer Bošković**
**MIC(µg/mL)**			
Staphylococcus aures, ATCC 29213 (QC)	**0,5-2**	1	>32
Staphylococcus aures, ATCC 43300 (MRSA)	**NA**	1	>32
Streptococcus pneumoniae, ATCC 49619 (QC)	**0,12-0,5**	0,125	>32
Enterococcus faecalis, ATCC 29212 (QC)	**1-4**	2	32

References

1. a) Hess, S.; Maier, M. E. Approach to the Core Structure of Streptosetin A. ChemistrySelect, **2020**, 5, 7315–7319. DOI: 10.1002/slct.202001018.
   b) Lacharity, J. J.; Fournier, J.; Lu, P.; Mailyan, A. K.; Herrmann, A. T.; Zakarian, A. Total Synthesis of Unsymmetrically Oxidized Nuphar Thioalkaloids via Copper-Catalyzed Thiolane Assembly. Journal of the American Chemical Society, **2017**, 139, 13272–13275. DOI: 10.1021/jacs.7b07685.
   c) Collins, D.; James, A. Preparation of 2-(3-Bromo-1-Methylpropyl)-1,3-Dioxolan and the Corresponding Chloride From 2-Methylbutyrolactone. Australian Journal of Chemistry, **1989**, 42, 223. DOI: 10.1071/ch9890223.

2. Rigaku Oxford Diffraction (2018). CrysAlisPro. Version 41.64.93a. Rigaku Oxford Diffraction, The Woodlands, Texas, USA.
3. G. M. Sheldrick, *Acta Cryst.*, **2015**, A71, 3.
4. G. M. Sheldrick, *Acta Cryst.*, **2015**, C71, 3.
5. O. V. Dolomanov, L. J. Bourhis, R. J. Gildea, J. A. K. Howard and H. Puschmann, *J. Appl. Cryst.*, **2009**, 42, 339.
6. C. R. Groom, I. J. Bruno, M. P. Lightfoot and S. C. Ward, *Acta Cryst.*, **2016**, B72, 171

NMR spectra

^{1}H NMR (600 MHz, $CDCl_3$)

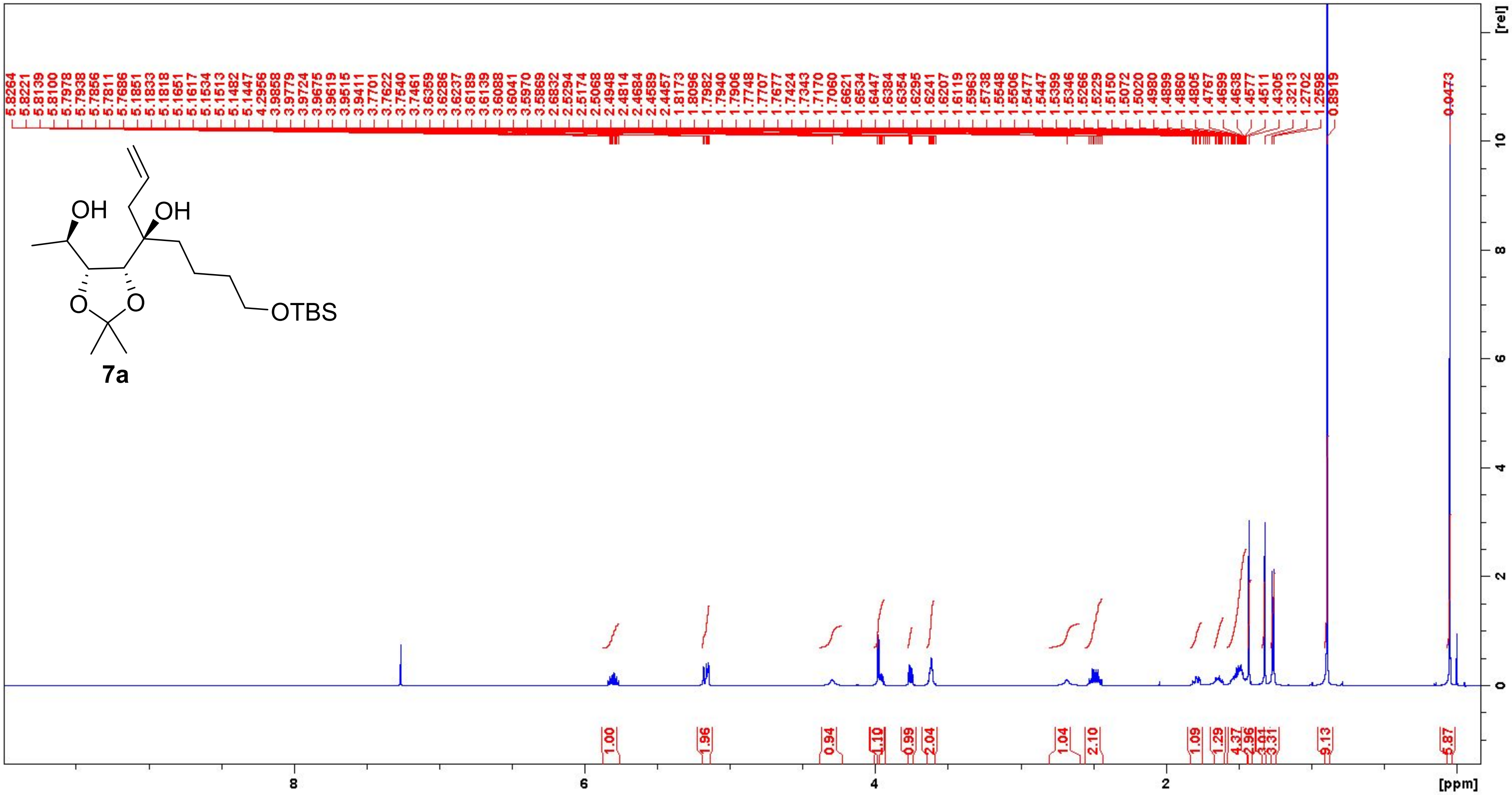

$^{13}C\{^{1}H\}$ NMR (151 MHz, $CDCl_3$)

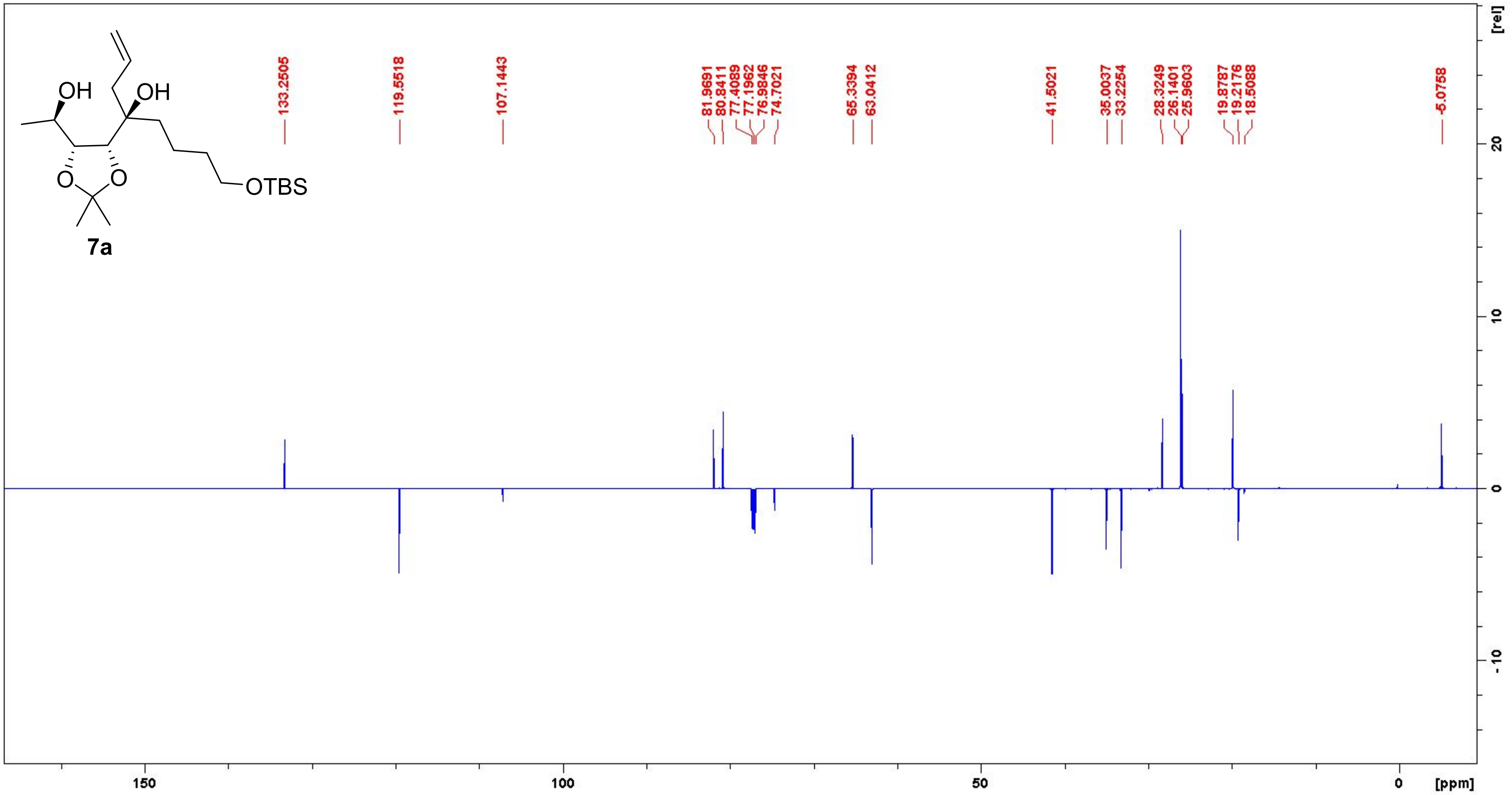

^1H NMR (400 MHz, $CDCl_3$)

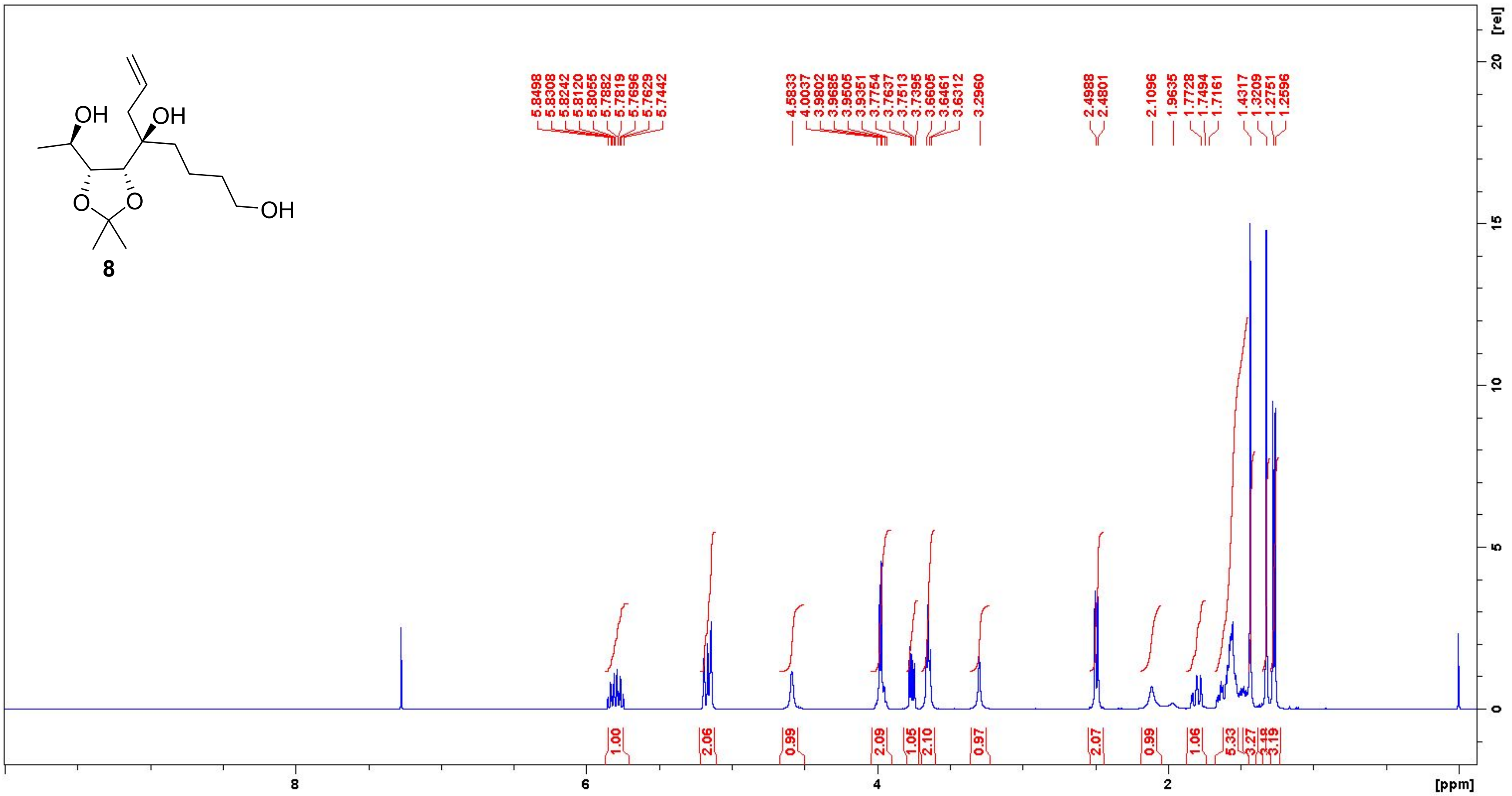

$^{13}C\{^1H\}$ NMR (100 MHz, $CDCl_3$)

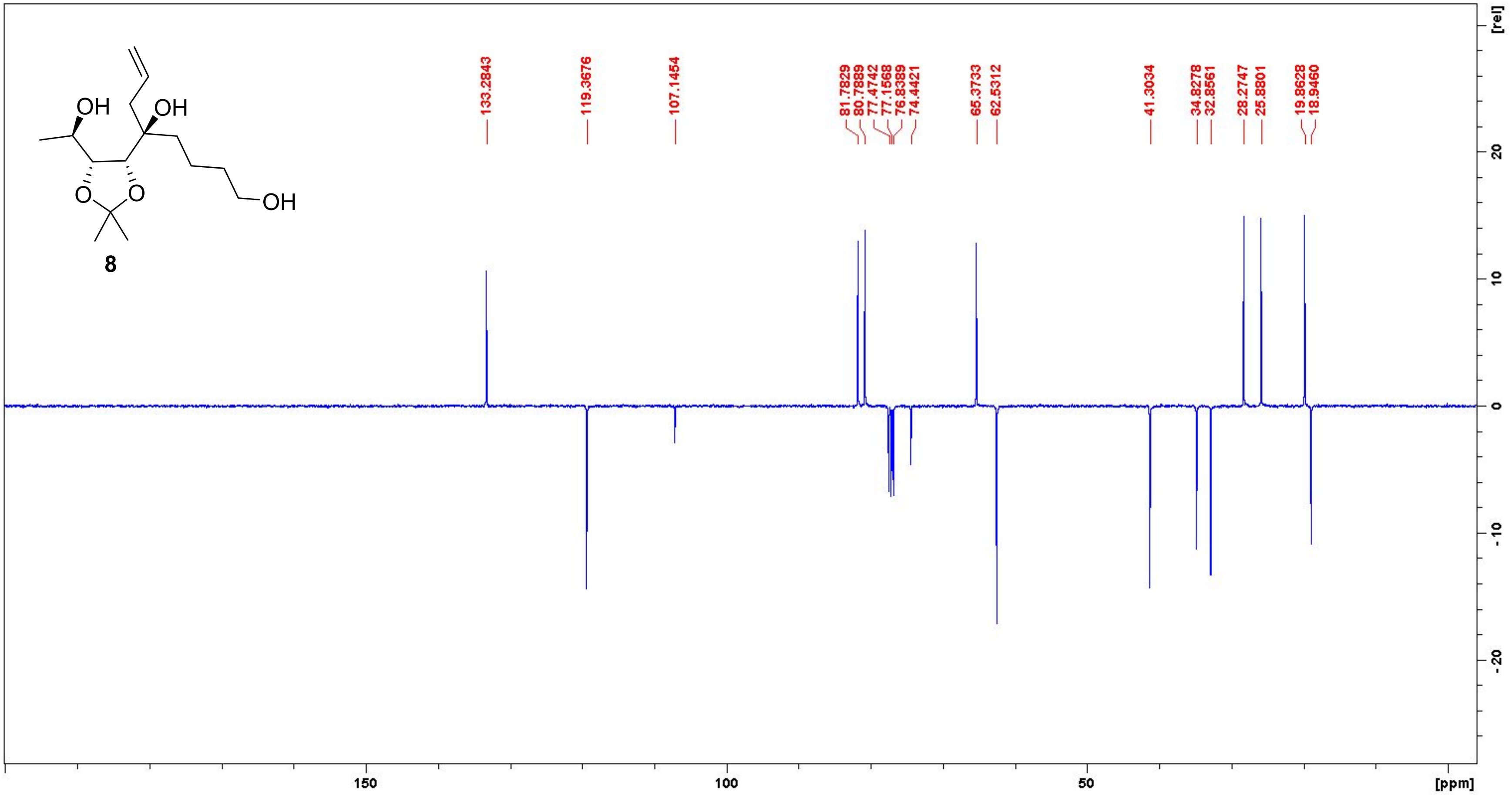

^{1}H NMR (600 MHz, $CDCl_3$)

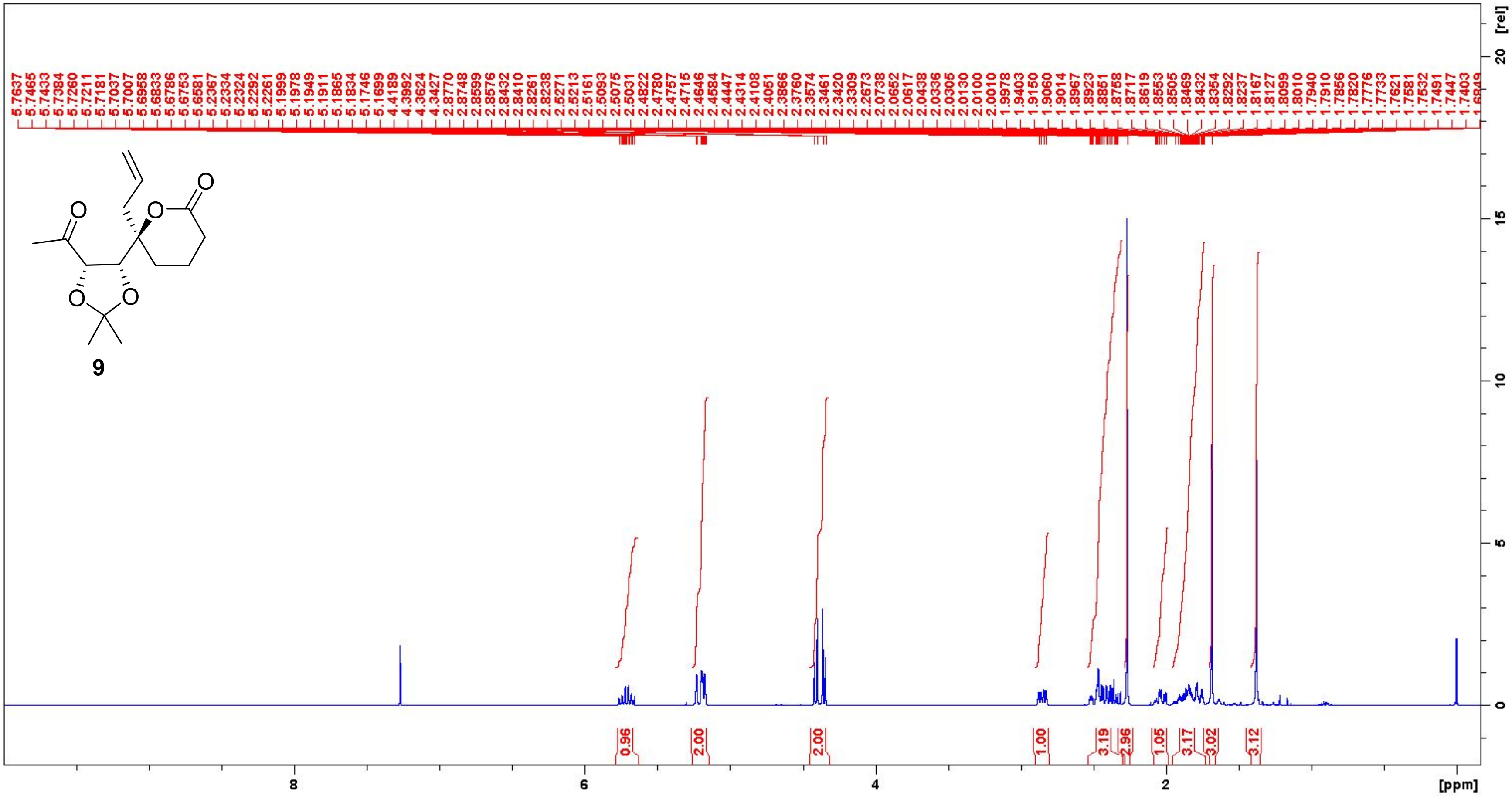

$^{13}C\{^{1}H\}$ NMR (151 MHz, $CDCl_3$)

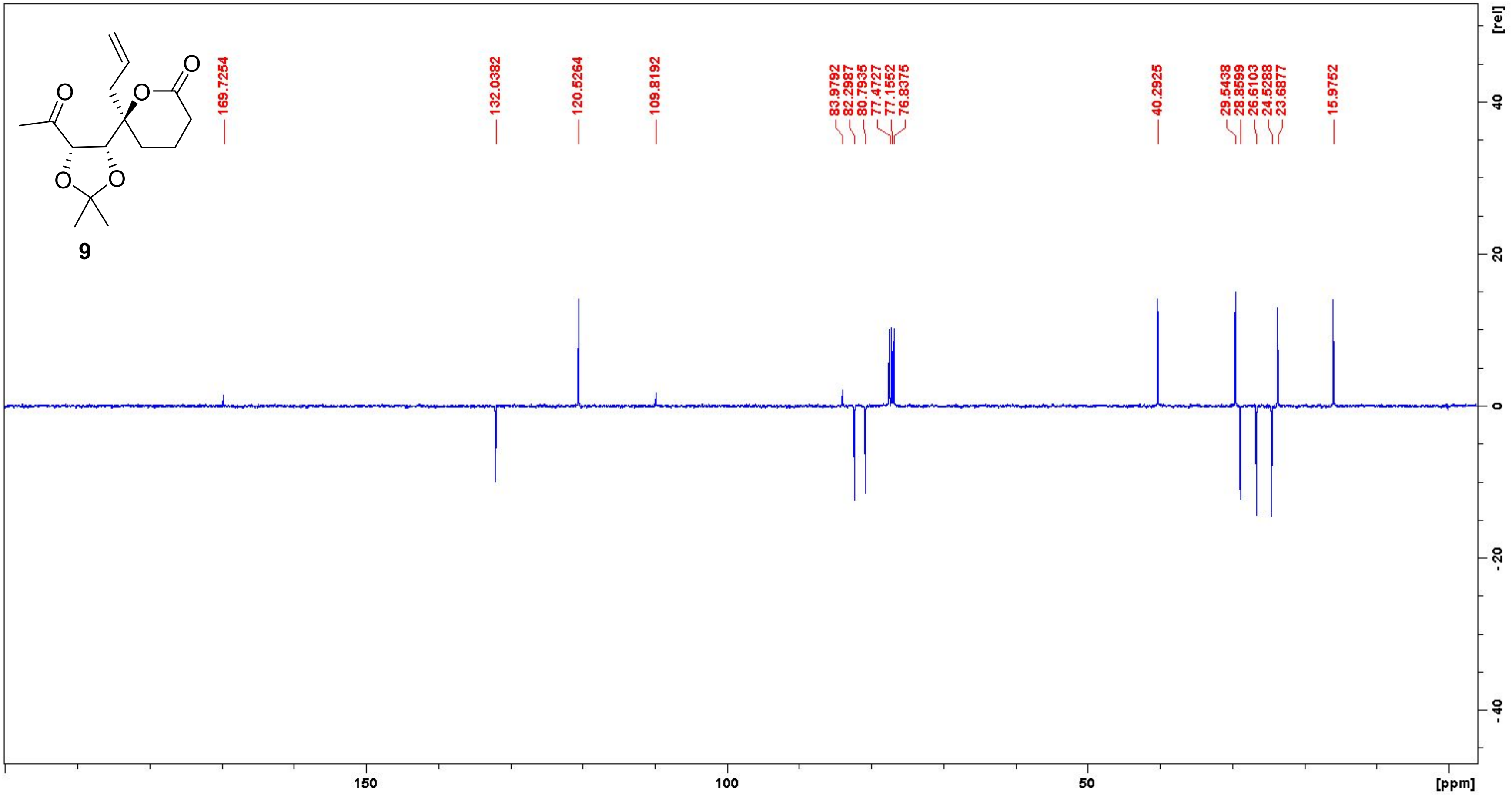

^{1}H NMR (400 MHz, $CDCl_3$)

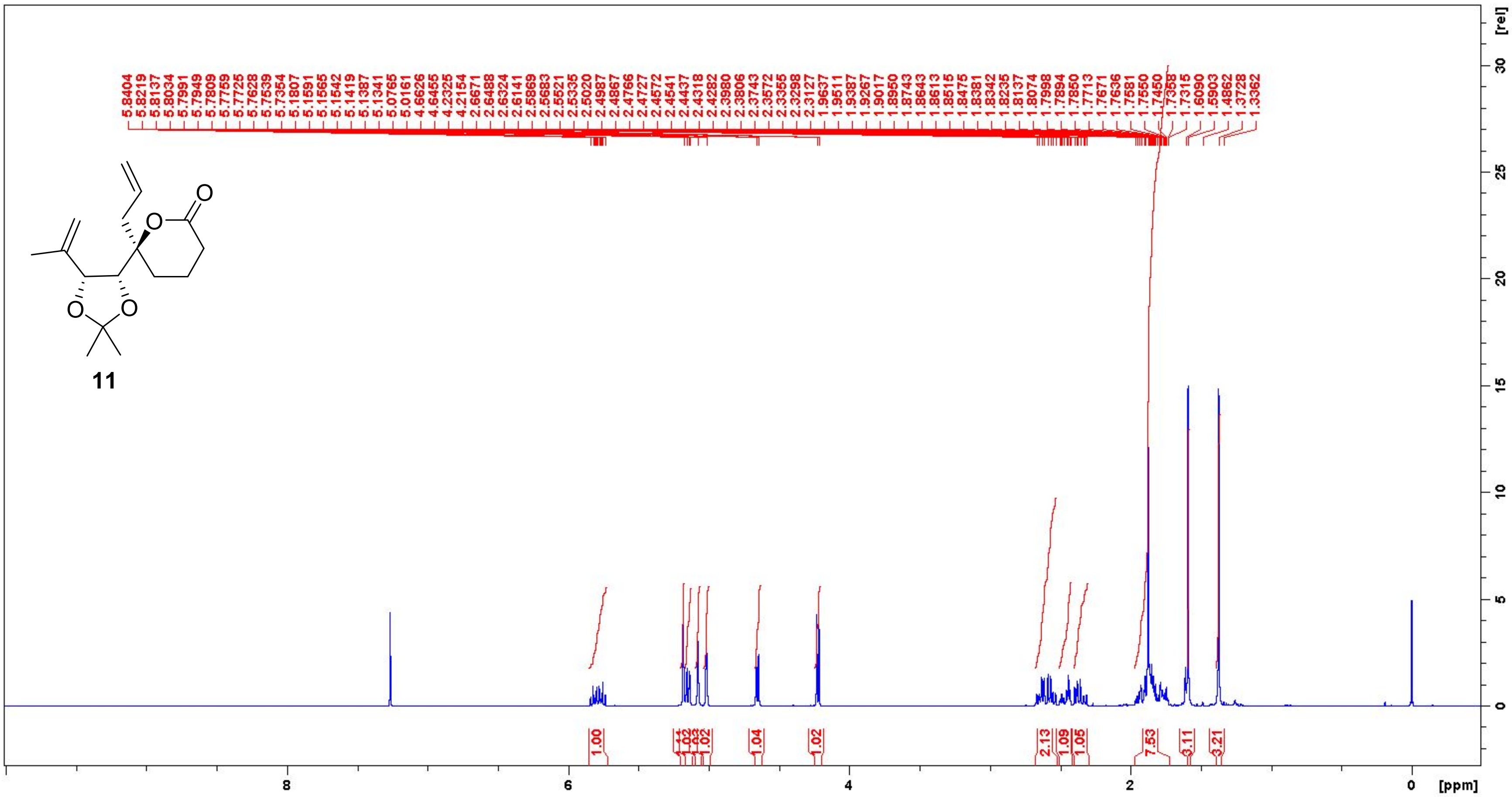

11

$^{13}C\{^1H\}$ NMR (100 MHz, $CDCl_3$)

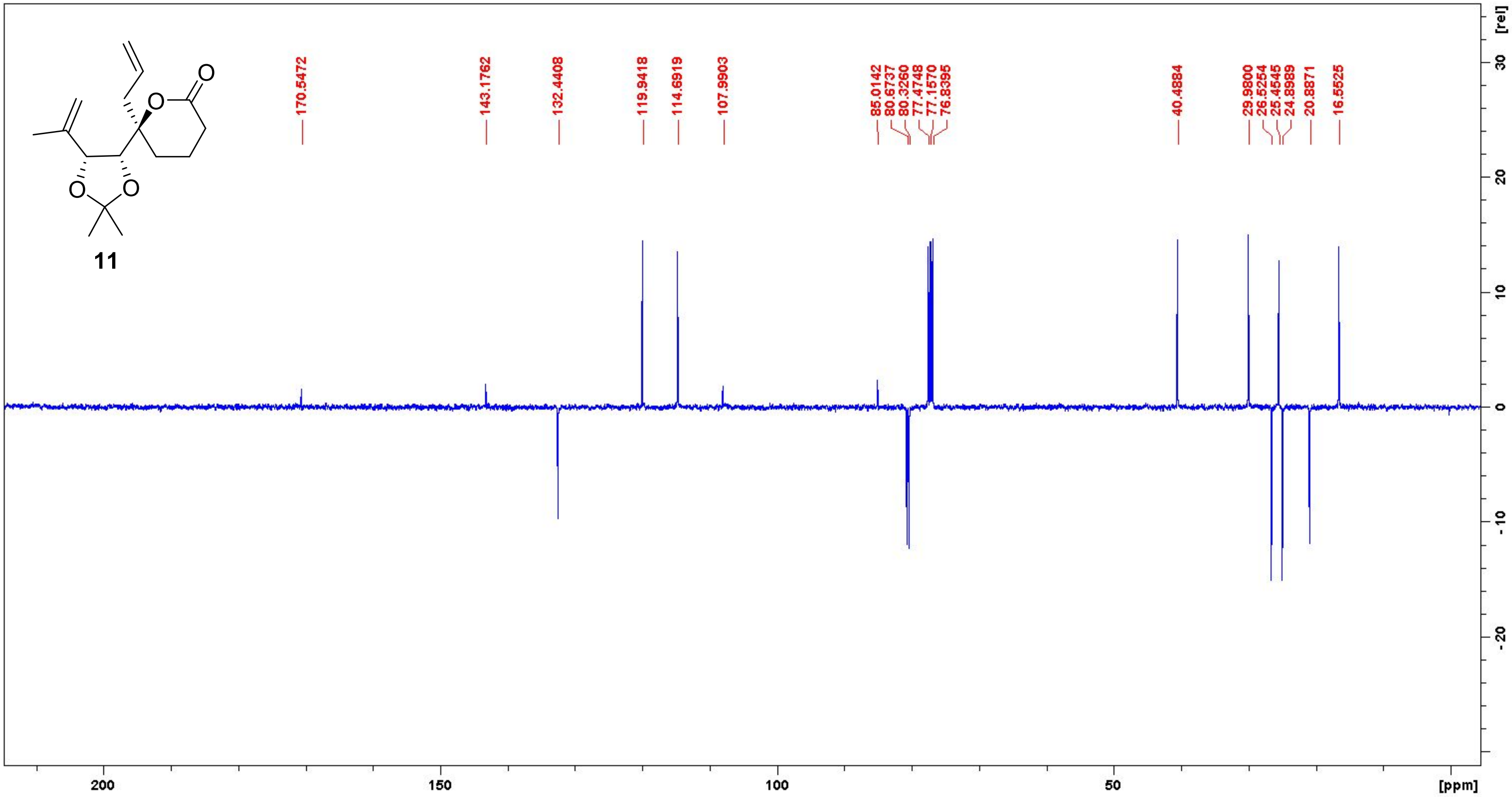

^{1}H NMR (600 MHz, $CDCl_3$)

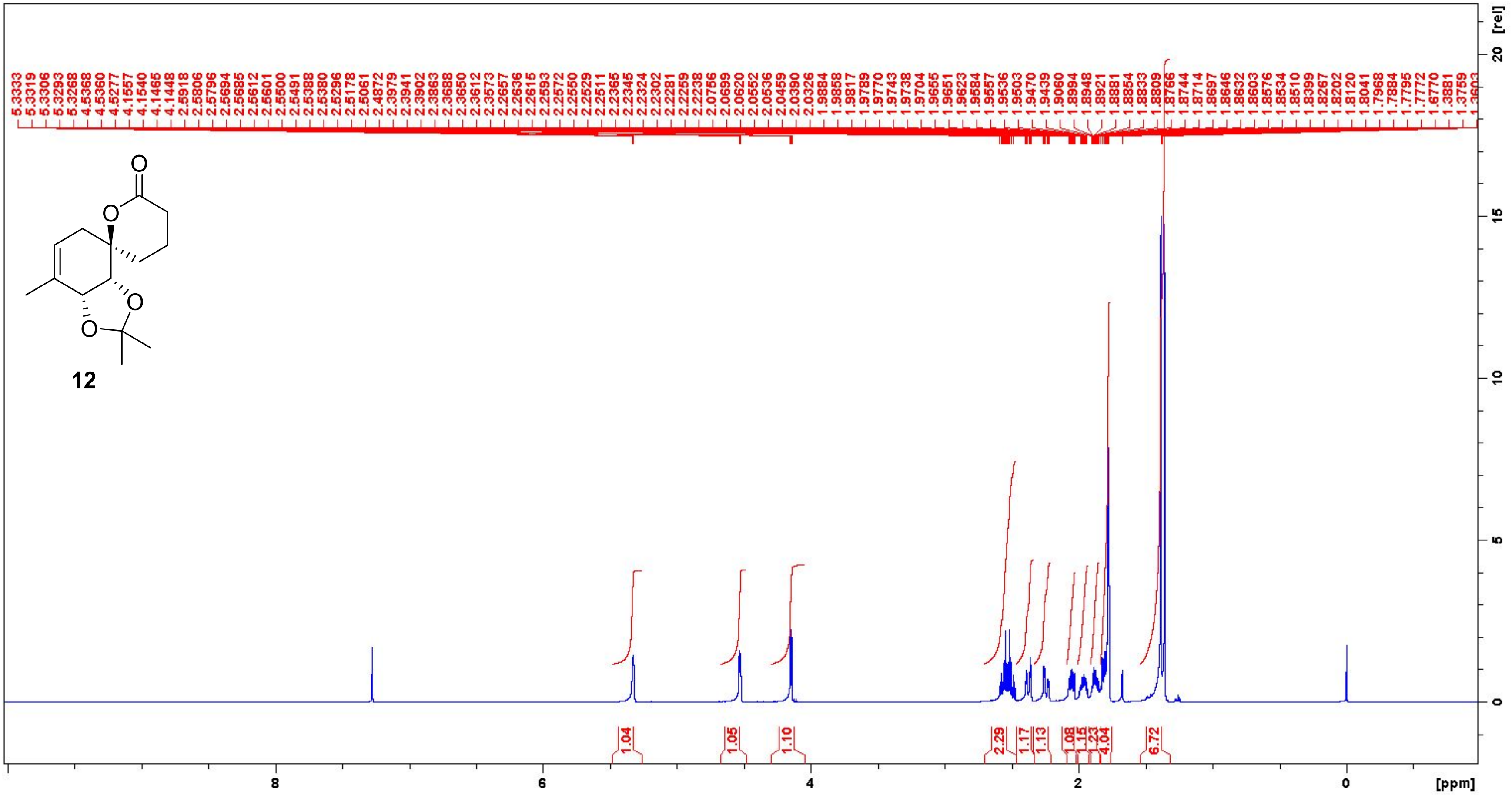

12

$^{13}C\{^{1}H\}$ NMR (151 MHz, $CDCl_3$)

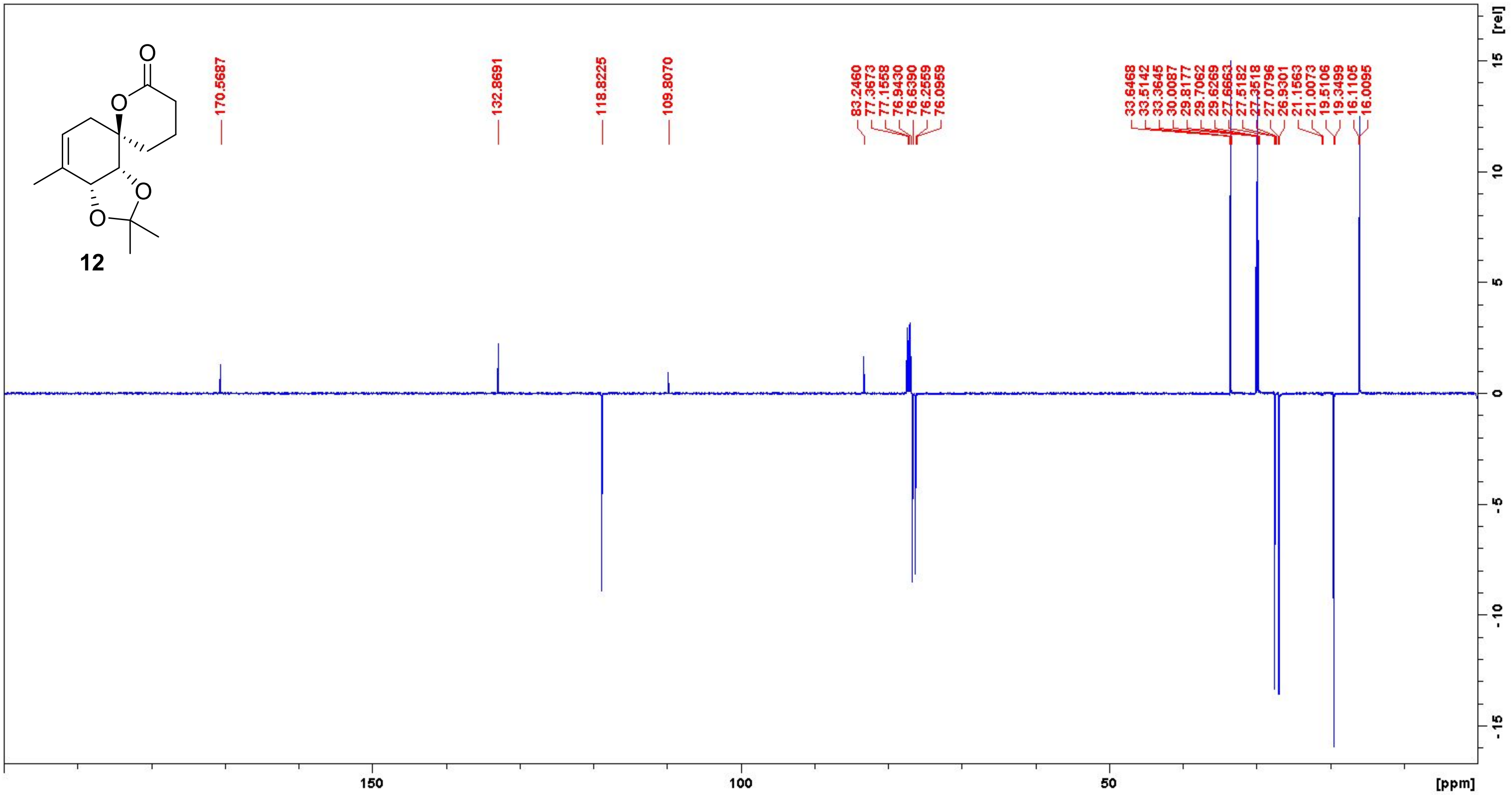

^1H NMR (600 MHz, $CDCl_3$)

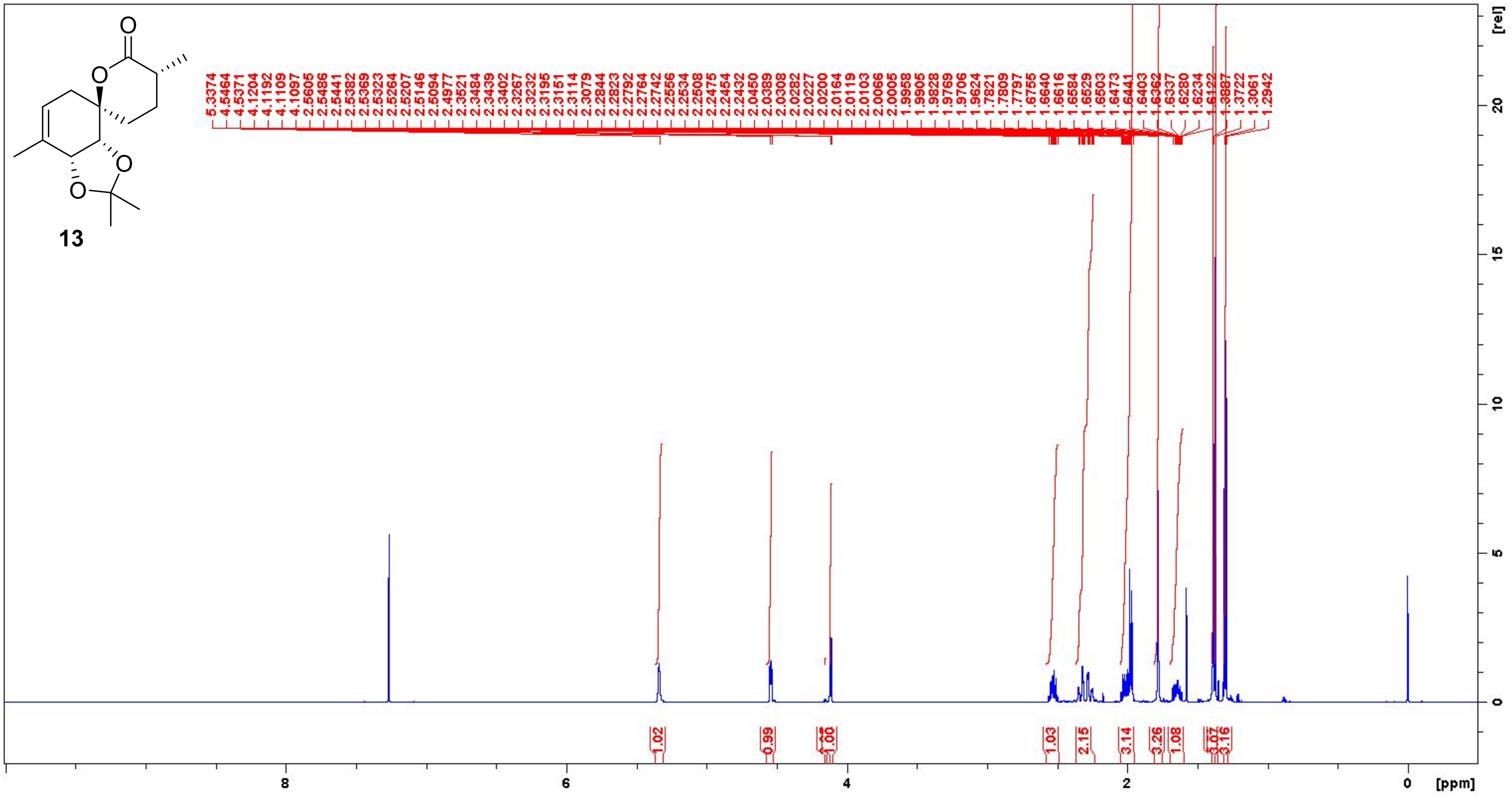

$^{13}C\{^1H\}$ NMR (151 MHz, $CDCl_3$)

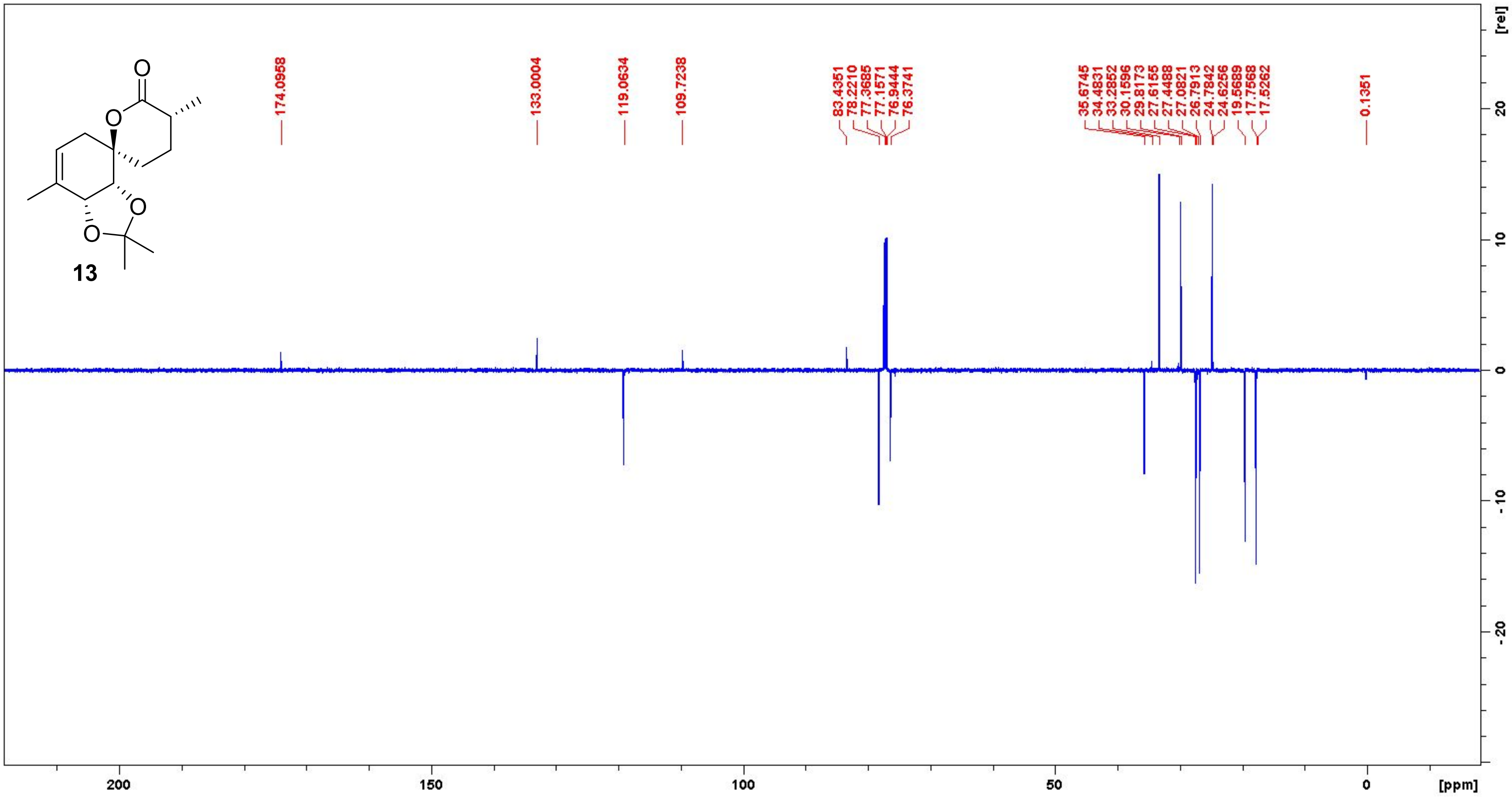

^{1}H NMR (600 MHz, $CDCl_3$)

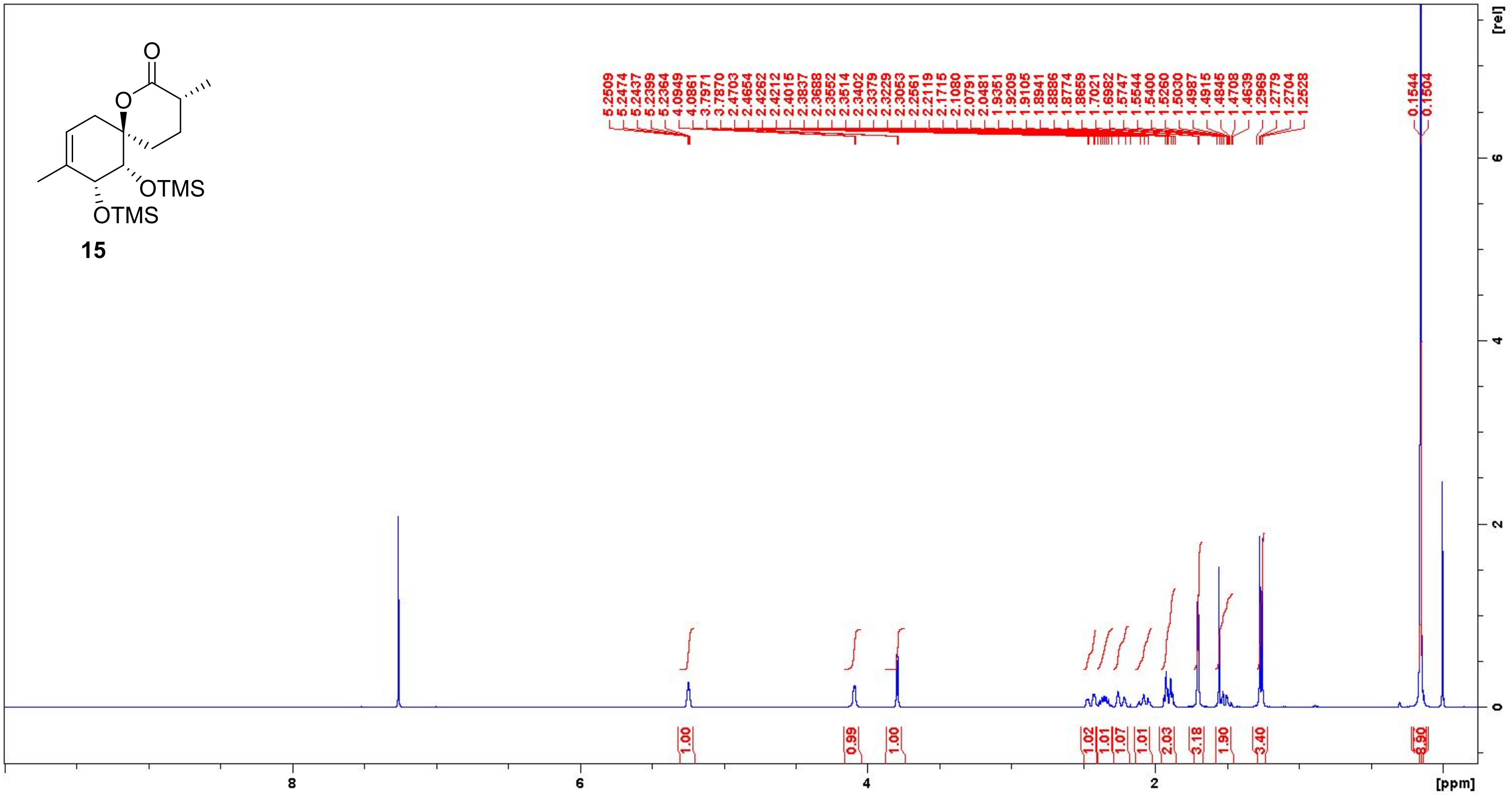

15

$^{13}C\{^{1}H\}$ NMR (151 MHz, $CDCl_3$)

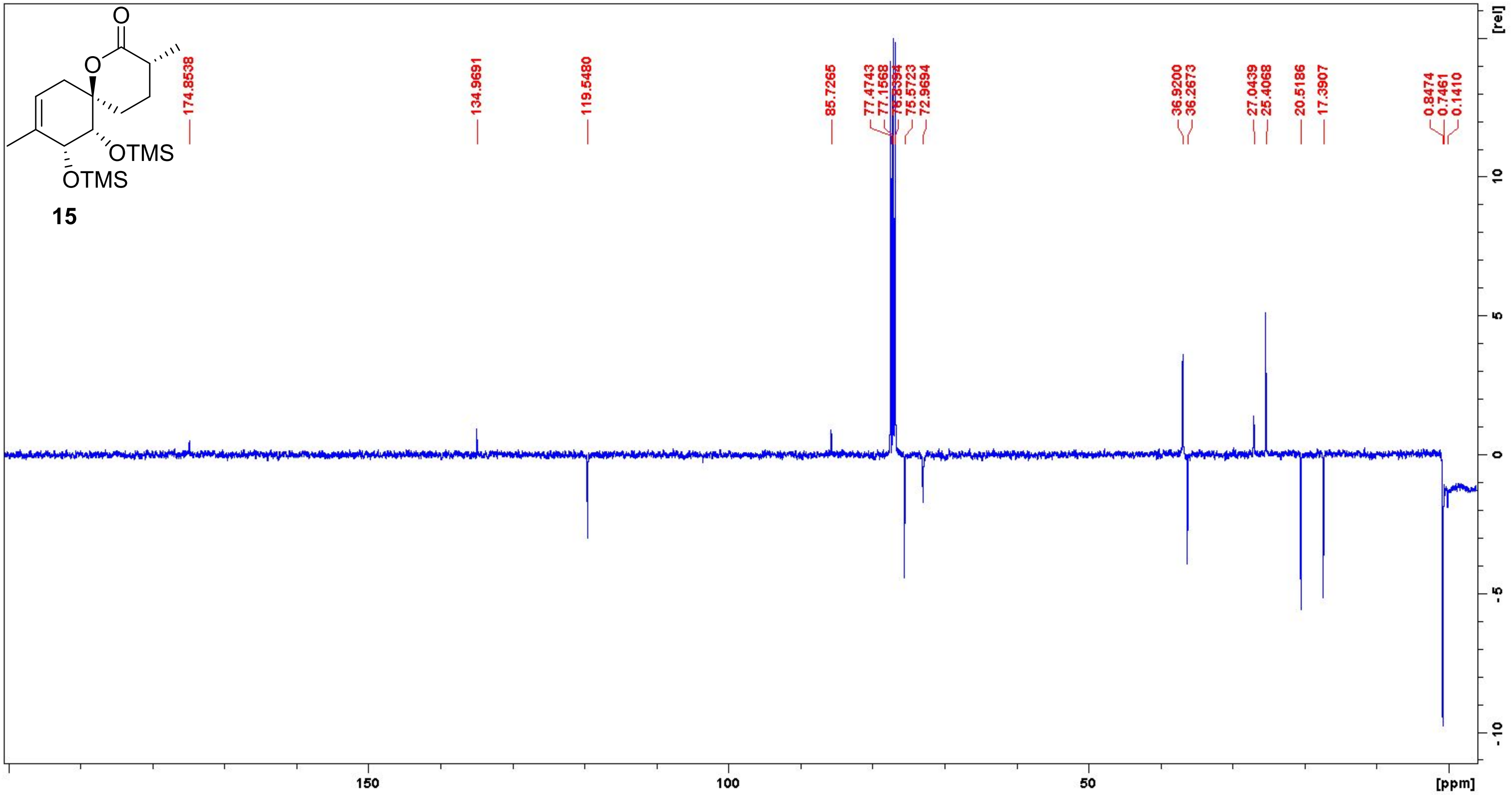

^{1}H NMR (400 MHz, $CDCl_3$)

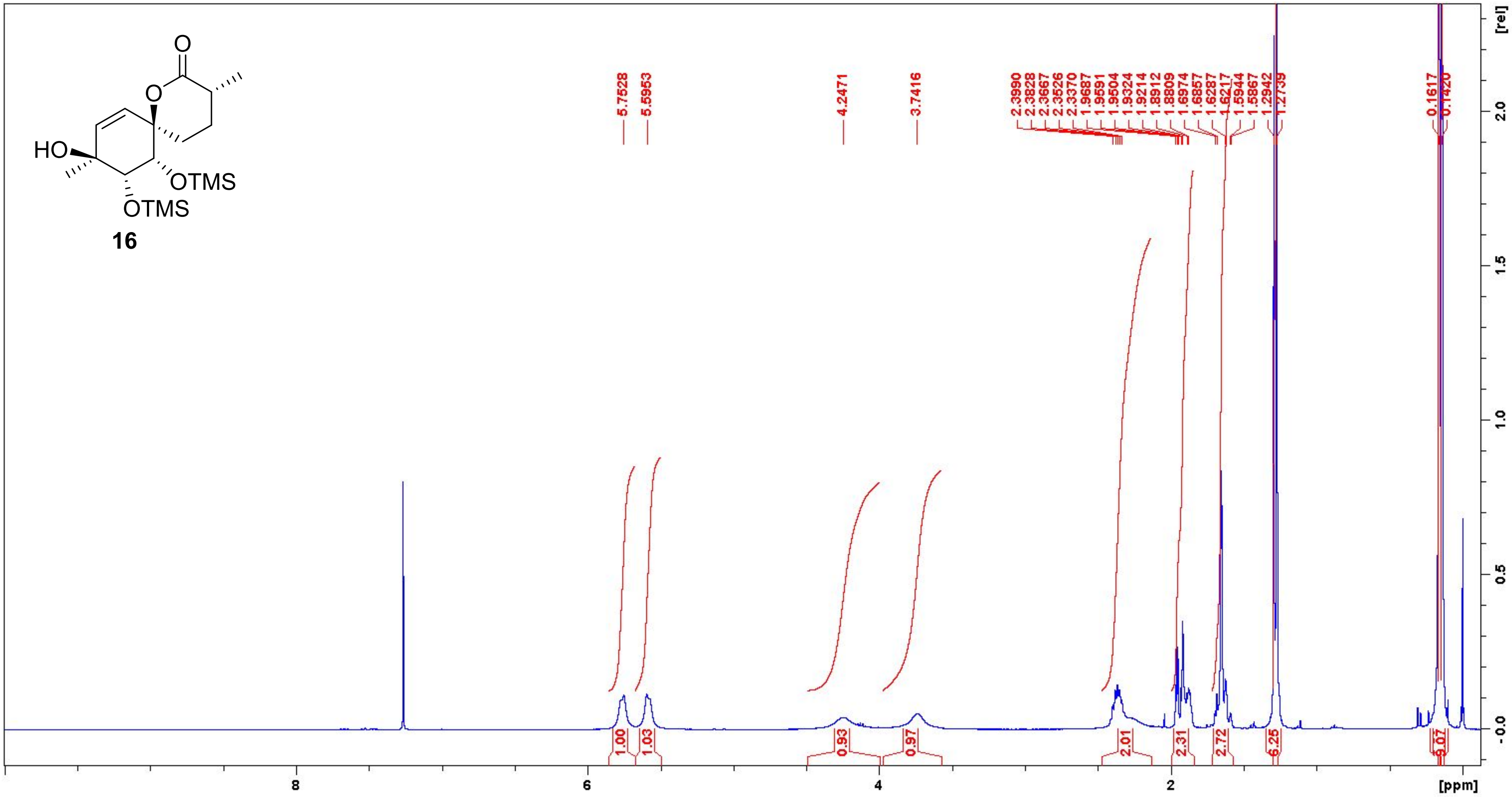

^{1}H NMR (600 MHz, $CDCl_3$)

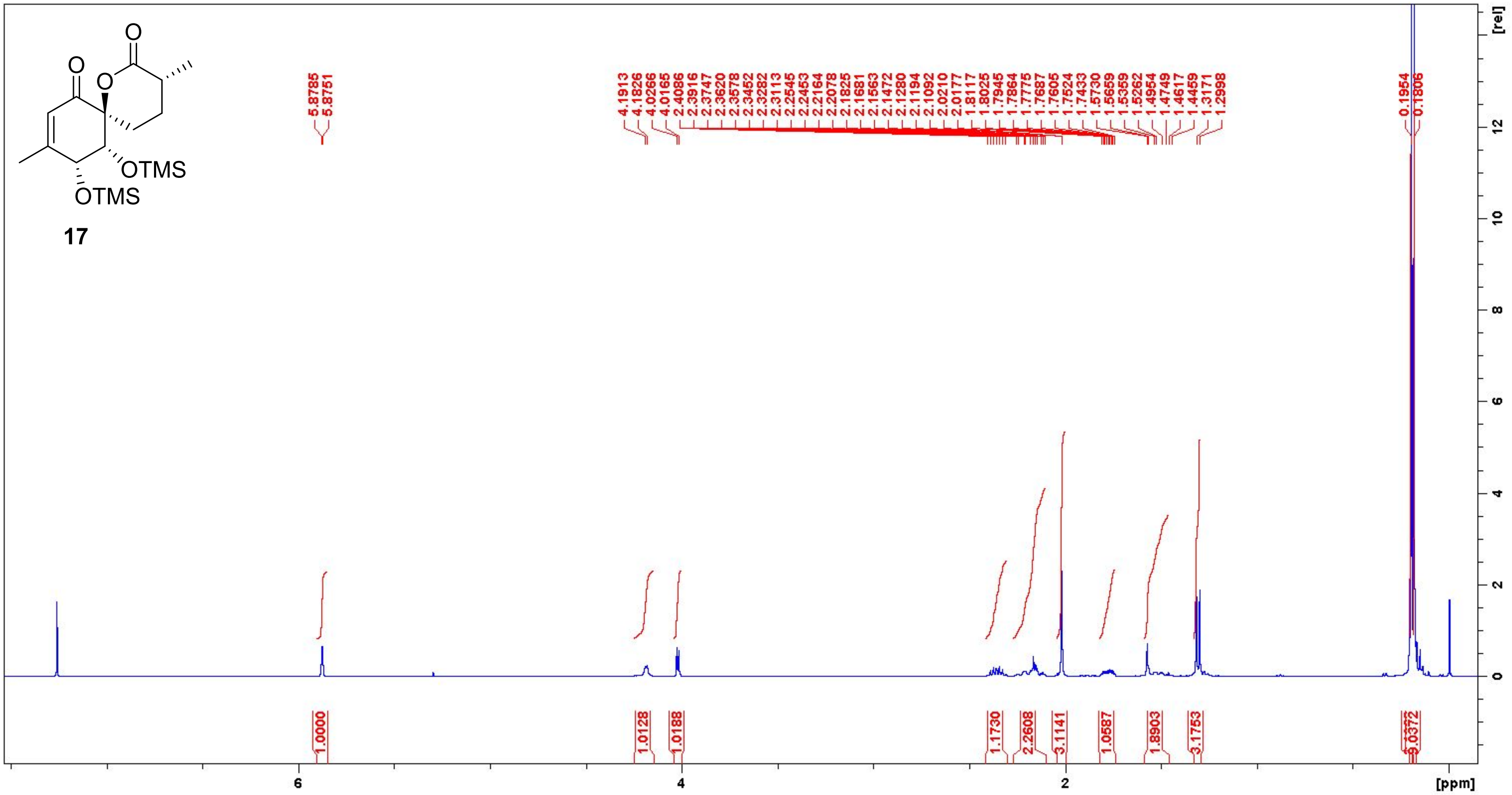

$^{13}C\{^{1}H\}$ NMR (151 MHz, $CDCl_3$)

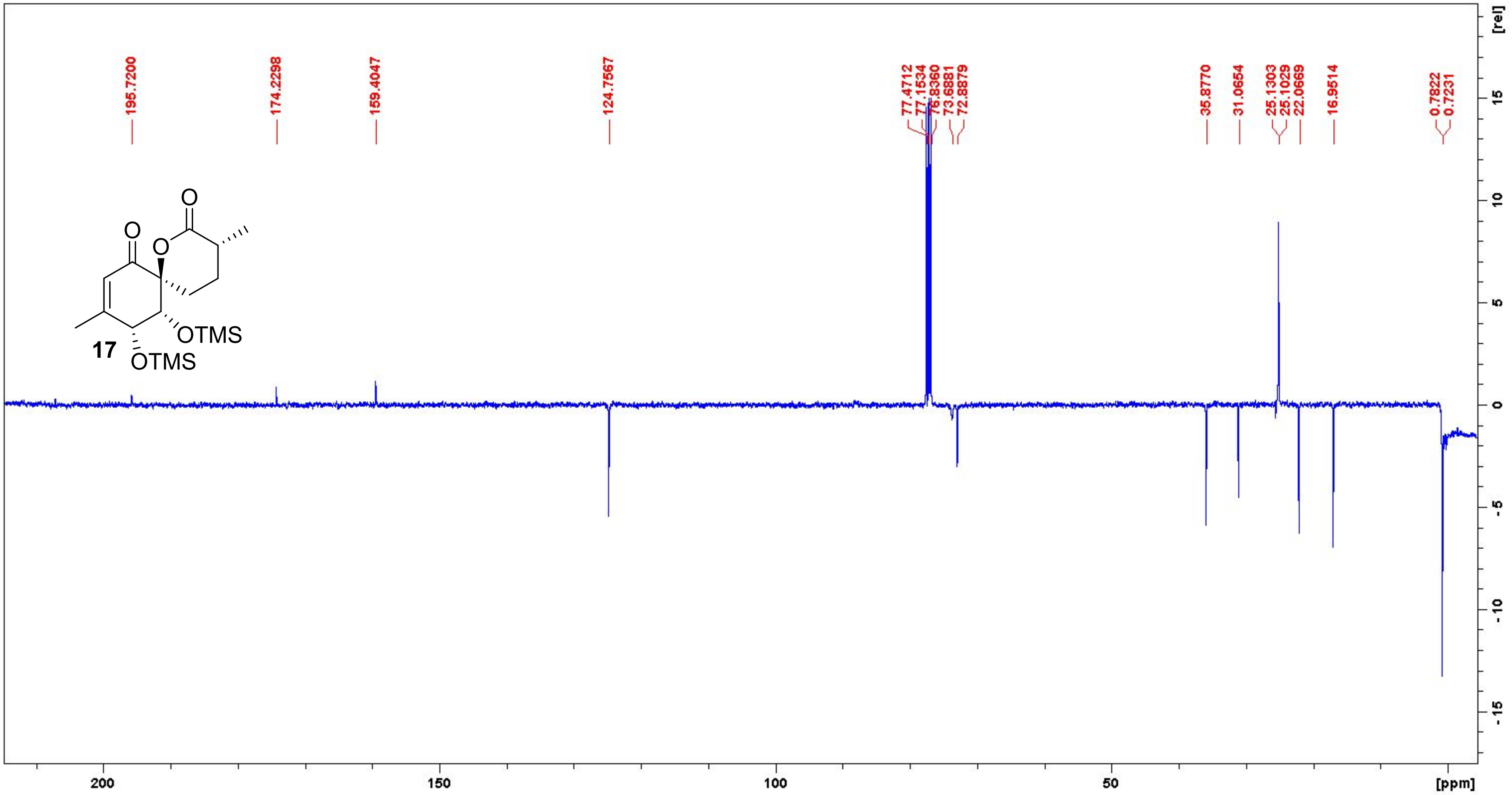

^{1}H NMR (600 MHz, DMSO-*d6*)

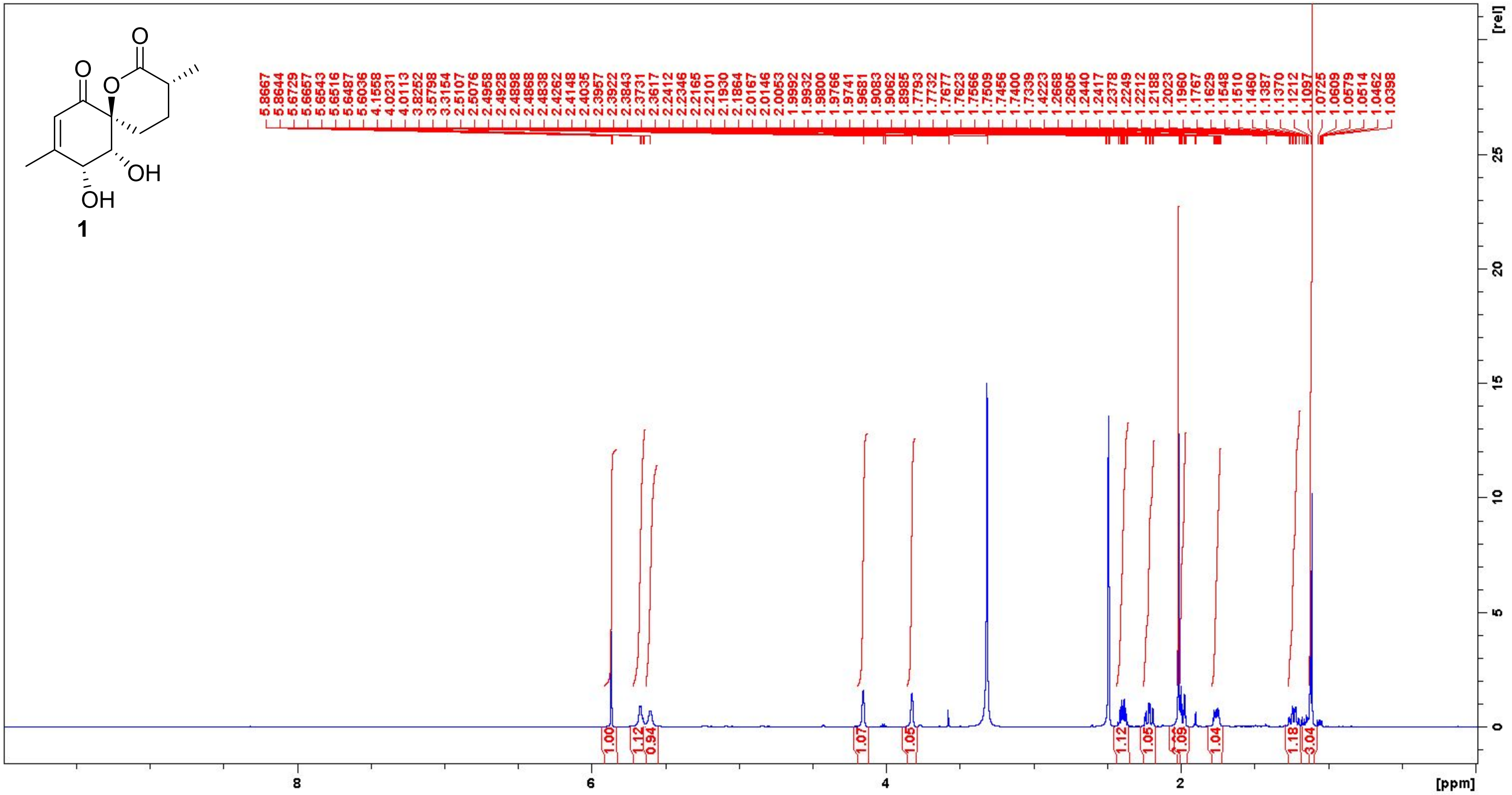

$^{13}C\{^1H\}$ NMR (151 MHz, DMSO-*d6*)

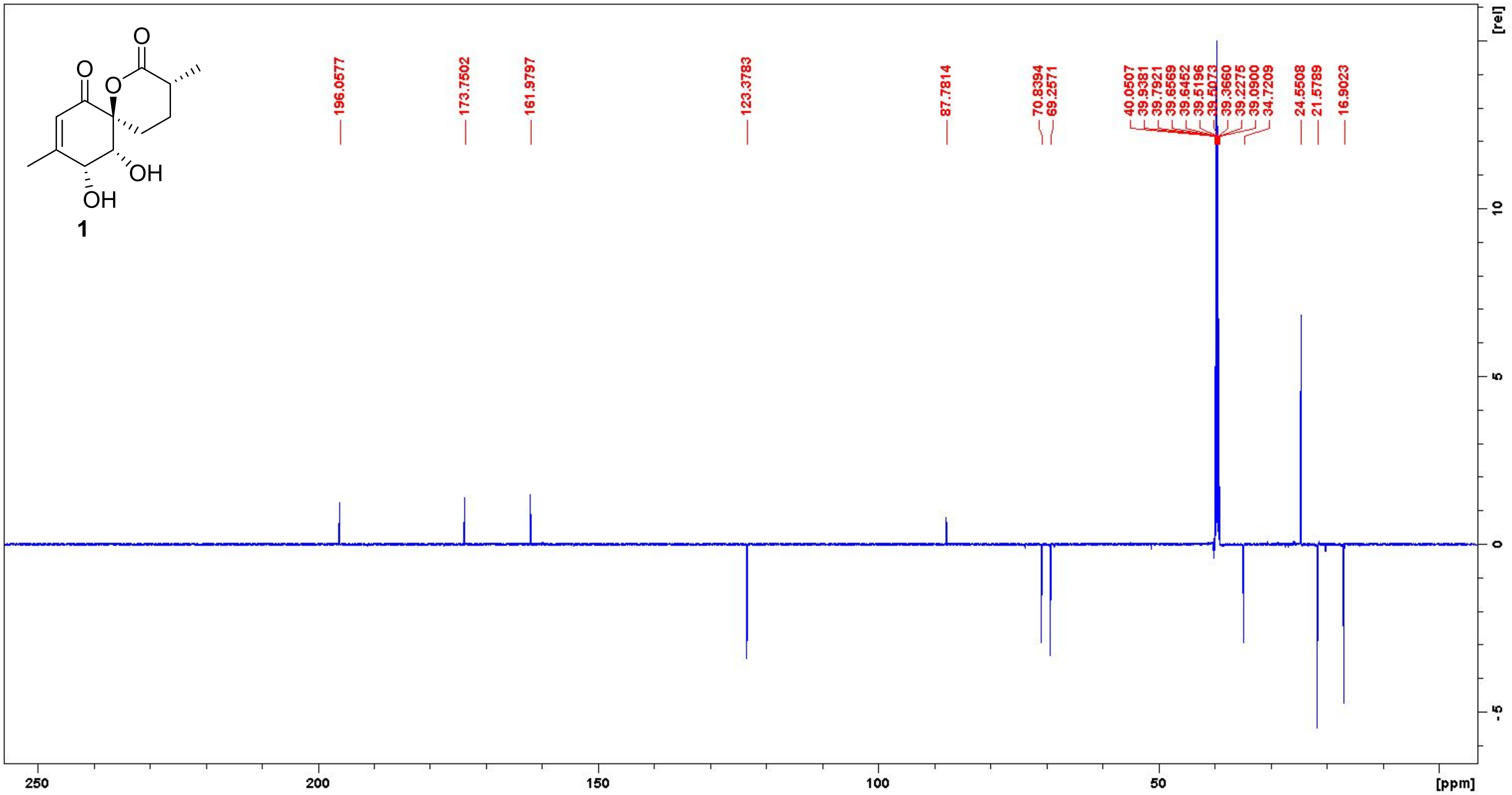

Supplement: Supplementary file 1 — jo2c02200_si_001.pdf [file jo2c02200_si_001.pdf]
